# Tumor hypoxia: Classification, detection, and its critical role in cancer progression

**DOI:** 10.17305/bb.2025.12318

**Published:** 2025-04-30

**Authors:** Yan Zhao, Bing Zhong, Jing-Yi Cao, Qian Wang, Jie Liang, Ke Lu, Sheng-Ming Lu

**Affiliations:** 1 Department of Urology, Xuzhou Cancer Hospital, Affiliated Hospital of Jiangsu University, Jiangsu, China; 2 Department of Urology, Xuzhou New Health Geriatric Disease Hospital, Jiangsu, China; 3 Department of Urology, The First Affiliated Hospital of Soochow University, Suzhou, Jiangsu, China; 4 Department of Urology, The First People’s Hospital of Huaian, Nanjing Medical University, Jiangsu, China; 5 Department of Urology, Changshu Second People’s Hospital, Yangzhou University Fifth Clinical Medical College, Jiangsu, China; 6 Department of Urology, Northern Jiangsu People’s Hospital Affiliated to Yangzhou University, Jiangsu, China

**Keywords:** Tumor, hypoxic, factors, classification, progression

## Abstract

Hypoxia is a common feature of solid tumors and plays a critical role in cancer progression. A thorough understanding of tumor hypoxia is essential for gaining deeper insights into various aspects of cancer biology. This review examines the key factors contributing to tumor hypoxia, such as inadequate blood supply and oxygen delivery resulting from rapid tumor growth. We present a detailed classification of hypoxic regions and provide an overview of current methods used to identify these areas—from molecular techniques to imaging approaches—offering a comprehensive and multifaceted perspective. Additionally, we explore the mechanisms by which hypoxia drives tumor progression. Under low-oxygen conditions, tumor cells can alter their biological behavior, influencing processes, such as cell proliferation, immune evasion, and the maintenance of tumor stem cells. By addressing these dimensions, we aim to enhance understanding of how hypoxia contributes to cancer development. Through this in-depth exploration, we hope this review will offer valuable insights to guide future research and clinical applications.

## Introduction

Solid tumors, formed by the uncontrolled proliferation of abnormal cells, pose a serious threat to human health. The compression of surrounding tissues and the potential for metastasis can lead to organ dysfunction and life-threatening conditions [1, 2]. Tumor cells disseminate through the blood and lymphatic systems, giving rise to distant metastases and complicating treatment efforts [3]. Some tumors exhibit high malignancy, characterized by rapid growth and poor treatment response, which imposes significant burdens on patients. In the United States, cancer ranks as the second leading cause of death, following heart disease; for individuals aged 60 and older, it is the leading cause of mortality [4]. Research indicates that the tumor microenvironment (TME)—comprising surrounding cells, stroma, and molecular components—plays a crucial role in tumor formation and progression, significantly influencing the success of cancer treatment strategies [5]. Within the TME, cellular components mainly include tumor cells, immune cells, fibroblasts, and endothelial cells [6]. The complex interactions among these cells, mediated by intricate signaling pathways, significantly influence tumor behavior. The extracellular matrix (ECM), composed of collagen, fibronectin, and various growth factors, also serves as a key structural component supporting tumor growth and spread [7]. Furthermore, tumors often induce angiogenesis (the formation of new blood vessels) to meet their high metabolic demands, a hallmark feature of the TME [8]. The complexity and diversity of the TME substantially increase the challenges of treating tumors. Hypoxia—a defining characteristic of the TME—is widespread in solid tumors and results from the imbalance between increased oxygen consumption and insufficient oxygen supply [9]. Normally, oxygen pressure in tissues exceeds 5.3 kPa, but in tumor tissues, it can fall to 0.9 kPa or lower [10]. This pervasive hypoxia stems from the uncontrolled growth, altered metabolism, and incomplete, inefficient architecture of tumors [11]. Hypoxia triggers changes in gene expression and proteomic profiles, profoundly impacting various cellular and physiological functions, ultimately worsening patient prognosis [12]. For example, cells in hypoxic regions, which tend to divide slowly, may evade the effects of cytotoxic drugs targeting rapidly dividing cells. Additionally, poorly oxygenated regions may harbor cancer stem cells (CSCs), promoting processes such as epithelial-to-mesenchymal transition (EMT) [13]. Overall, a deeper understanding of hypoxia in the TME can aid in developing novel treatment strategies, enhancing the effectiveness of cancer therapies, and improving patient outcomes. Therefore, this study focuses on investigating the factors influencing hypoxia, its classification, the identification of hypoxic regions, and the role of hypoxia in promoting tumor progression.

### Introduction to tumor hypoxia

#### Factors leading to tumor hypoxia

The internal hypoxia within tumors is an intricately complex phenomenon influenced by multiple factors. First, the rapid proliferation of tumor cells far exceeds the rate of vascular network formation, leading to the development of avascular hypoxic regions within the solid tumor. This creates a challenging environment for tumor cell survival and proliferation [9]. Additionally, solid tumors are characterized by a convoluted vascular system that is structurally and functionally abnormal, with features, such as tortuous vessels, erratic growth, and defective endothelial cells. These abnormalities result in uneven blood supply, exacerbating hypoxic conditions and further complicating the already arduous task of sustaining tumor cell viability in this harsh microenvironment [14, 15]. Research has shown that newly formed solid tumors initially lack the ability to generate their own blood vessels and instead rely on the diffusion of oxygen from nearby host vasculature to support their growth and proliferation [16]. As these avascular tumor masses expand, the increasing distance from the central regions to the nearest blood vessels inevitably leads to hypoxia [17, 18]. Moreover, the difficulties associated with transporting essential substances between vessels further aggravate the problem [19]. The high oxygen demand of rapidly proliferating tumor cells intensifies cellular competition for oxygen, leading to a noticeable increase in consumption. This competition broadens the scope of hypoxic regions, forming a vicious cycle that makes it increasingly difficult for tumor cells to maintain normal physiological activities in hypoxic environments [20]. Chemotherapy and radiation therapy can also contribute to tumor hypoxia. Research by Mizrachi et al. [21] explored the vascular damage induced by the chemotherapy drug doxorubicin (DOX) using both *in vitro* and *in vivo* approaches. *In vitro* experiments demonstrated that DOX significantly increases the activity of acid sphingomyelinase (ASMase), leading to the generation of reactive oxygen species (ROS) and subsequent cell apoptosis. *In vivo*, molecular imaging techniques revealed that DOX caused constriction of small vessels and destruction of large vessel walls in the femoral artery of mice. These findings suggest that DOX primarily triggers ROS production through the ASMase pathway, resulting in endothelial cell apoptosis and acute vascular injury. Additionally, there is compelling evidence that angiopathy is a common complication following radiation therapy (RT) due to ensuing vascular damage. In both adult and pediatric patients, this condition can manifest in several ways, including steno-occlusive changes in blood vessels, intracranial hemorrhages, aneurysm development, and other vascular abnormalities [22]. The vascular damage caused by radiotherapy and chemotherapy not only increases the likelihood of vascular malformations but also diminishes the stability of the vascular system, leading to a more chaotic blood flow distribution. This instability makes it more challenging to achieve the intended therapeutic effects and may further exacerbate tumor hypoxia, posing even greater challenges to treatment.

**Figure 1.**
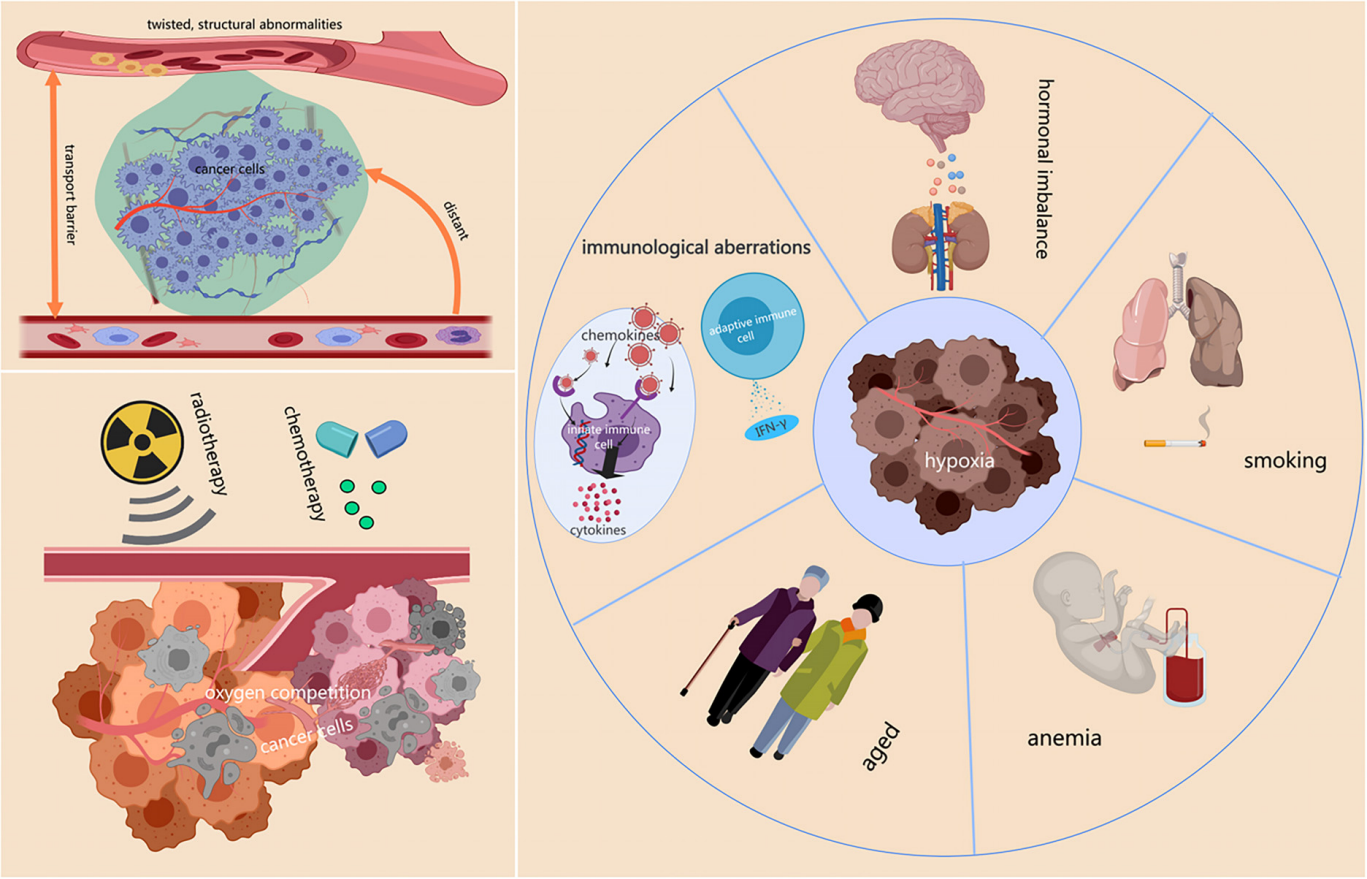
**Factors leading to tumor hypoxia.** The rapid proliferation of tumor cells far exceeds the formation speed of the surrounding vascular network, resulting in the formation of avascular hypoxic zones within solid tumors. The vascular system within solid tumors exhibits structural and functional abnormalities, including vascular distortion, abnormal proliferation, and dysfunction of endothelial cells. The distance between the tumor center and the nearest blood vessels increases, causing hindrance to the transport of substances between vessels. Intense competition among tumor cells leads to a significant increase in oxygen consumption. Factors such as radiation therapy, chemotherapy, anemia, advanced age, smoking, immunological abnormalities, and hormonal imbalances also contribute to tumor hypoxia.

Anemia, typically caused by disease states or treatment processes, is a pathological condition characterized by a decreased number of red blood cells or lower hemoglobin levels, compromising the blood’s oxygen-carrying capacity. This reduction in oxygen delivery leads to the formation of hypoxic regions, which adversely affect the functionality and survival of tumor cells [23, 24]. Research has further demonstrated that the blood’s oxygen-carrying capacity deteriorates with advancing age and chronic smoking, potentially exacerbating intratumoral hypoxic conditions [25, 26]. However, additional studies are needed to clarify the complex interactions between these systemic factors and their cumulative effects on the TME. Physiological factors, such as hormone levels, may also influence tumor hypoxia. Data suggest a link between estrogen and the hypoxic pathway, wherein estrogen-mediated signals can directly drive hypoxia-inducible factor-1α (HIF-1α) expression and modulate the hypoxic response, either positively or negatively, depending on the cellular environment [27]. Additionally, changes in immune system activity can trigger chronic inflammatory responses, increasing immune cell infiltration and disrupting normal blood vessel function. Immune cells intricately shape the phenotype and function of tumor vessels through the interplay of various cytokines. Innate immune cells, such as mature dendritic cells (mDCs) and M1-like tumor-associated macrophages (TAMs), secrete cytokines, such as IFN-α, IL-12, IL-18, and TNF, along with chemokines like CXCL9, CXCL10, and CCL21, which effectively suppress tumor angiogenesis. Similarly, adaptive immune cells, including CD8+ T cells and T helper 1 (TH1) cells, release IFN-γ, a potent cytokine that not only inhibits angiogenesis but also promotes vascular normalization within the TME [28]. These immune-driven changes contribute to vascular malformations within tumors, ultimately leading to localized hypoxia. In summary, the explosive proliferation of tumor cells generates regions within the solid mass that suffer from insufficient blood supply. Known as hypoxic zones, these regions arise from the increased distance between cells and functional blood vessels, abnormalities in the tumor vasculature’s structure and function, and the intense competition for oxygen among rapidly dividing cells. External factors, such as radiotherapy, chemotherapy, anemia, advanced age, smoking, immune dysregulation, and hormonal imbalances can further exacerbate tumor hypoxia (Figure 1). The detailed roles of these various influences are outlined in Table 1, which categorizes the factors affecting tumor hypoxia—such as cell proliferation, vascular abnormalities, treatments, blood-related issues, and physiological factors—along with their effects (e.g., outpacing vessel growth, impairing vascular function, and reducing oxygen-carrying capacity) and consequences (e.g., exacerbated tumor hypoxia). Understanding the intricate interplay among these factors underscores that tumor hypoxia is not the result of a single cause but rather the outcome of multiple interacting influences. This comprehensive understanding offers valuable insights for the development of more precise and effective therapeutic strategies.

**Table 1:** The detailed role of different factors affecting hypoxia and its consequences

**Factor**	**Influence**	**Consequence**
Rapid proliferation of tumor cells	Surrounding blood vessel growth outpaced	
Vascular system abnormality	Structural distortion, irregular growth, and functional defects lead to uneven blood supply	
Newly formed solid tumors are located far from blood vessels	Blood vessels provide poor oxygen supply to tumor tissues	
Oxygen demand and competition	Increased oxygen consumption	
Chemotherapy	Generate ROS, leading to apoptosis of endothelial cells and damage to tumor blood vessels	Tumor hypoxia
Radiation therapy	Tumor vascular abnormalities, such as vessel occlusion, rupture, and formation of aneurysms, etc.	
Anemia	A decrease in red blood cells or hemoglobin reduces the blood’s ability to carry oxygen	
Advanced age and smoking	A decrease in the ability of the blood to carry oxygen	
Changes in hormone levels	Effects on hypoxia factor expression and impact on hypoxia levels in varied environments	
Immune system changes	Immunocytes release substances that inhibit tumor angiogenesis, leading to vascular malformations	

### Classification of tumor hypoxia

Research reports suggest that the biological and therapeutic consequences of hypoxia vary depending on the type. Thus, distinguishing and quantifying these subtypes may be necessary [29]. Factors contributing to tumor hypoxia mainly include abnormal vascular structure and function within solid tumors, increased distances for substance transport between vessels, intense competition for oxygen among rapidly proliferating tumor cells, and disease- or treatment-induced anemia that reduces the blood’s oxygen-carrying capacity [30–32]. Based on these mechanisms and the duration of hypoxia, tumor hypoxia is primarily classified into three types: perfusion hypoxia, diffusion hypoxia, and anemic hypoxia [23, 33, 34]. Perfusion hypoxia, also known as acute hypoxia, typically lasts from minutes to hours during periods of oxygen deprivation [29]. It is primarily caused by temporary interruptions in blood flow, often due to physical obstructions in blood vessels [33, 35]. These transient interruptions can result from vascular thrombosis, vessel rupture, and hemodynamic changes. Vascular occlusion—caused by blood clots or tumor cell invasion—blocks blood flow and leads to sudden hypoxia in affected areas [36]. Tumor-induced hypoxia can also promote clot formation, creating a vicious cycle [37, 38]. Vascular rupture, stemming from abnormal structure or fragility within tumor vessels, results in blood leakage and reduced perfusion. Furthermore, hemodynamic instability in tumors exacerbates hypoxia. Studies have shown that combining cediranib with radiotherapy and chemotherapy improves tumor perfusion in about 50% of glioblastoma patients, highlighting the potential of adjuvant therapies to affect tumor hemodynamics [39]. Nevertheless, once established, a hypoxic environment can further destabilize hemodynamics [40]. In summary, a combination of these factors can precipitate acute hypoxia. Diffusion-related hypoxia, or chronic hypoxia, develops during prolonged oxygen deprivation lasting from hours to weeks [29]. It is observed in 65%–86% of tumor tissues [33, 41]. Chronic hypoxia results from several factors, including the substantial distance between tumor cells and blood vessels (cells located more than 70 µm away are prone to oxygen deficiency); poor blood supply caused by structural abnormalities, such as vessel perforations, blunt ends, tortuosity, sluggish flow, and poorly perfused branches; increased intra-tumoral pressure from solid stress or interstitial fluid pressure; and changes in diffusion dynamics, such as differences between concurrent and countercurrent blood flow within the tumor microvessels. These factors together sustain reduced delivery of oxygen, nutrients, growth factors, and impede the transport of anticancer and imaging agents [33, 35, 42]. Anemic hypoxia, also known as systemic hypoxia, refers to oxygen deficiency in tissues due to insufficient oxygen-carrying capacity of the blood. This typically arises from a reduction in red blood cells and hemoglobin levels. Experimental studies show that tumor oxygen supply significantly diminishes, and hypoxia worsens when hemoglobin levels fall below 10–12 g/dL, especially when both oxygen transport capacity and perfusion are compromised [43, 44]. Therefore, managing anemia effectively is critical for improving the outcomes of other therapeutic interventions.

There are three main therapeutic approaches for addressing anemia: iron supplementation, blood transfusion, and the use of erythropoiesis-stimulating agents (ESAs). However, both transfusions and ESA administration carry a significant risk of severe adverse effects, particularly thromboembolism. Given that iron-deficiency anemia is a common cause of anemia in cancer patients, it is essential not only to assess hemoglobin levels but also to evaluate serum ferritin and transferrin saturation. In cases of absolute iron deficiency, iron supplementation is critical, while in instances of functional iron deficiency, supplementation should be complemented with ESA therapy [45, 46]. Some studies [47] suggest that increasing hemoglobin levels in patients with head and neck squamous cell carcinoma (HNSCC) may lead to adverse outcomes, including decreased survival and compromised tumor control. These unexpected results may be due to the stimulation of tumor growth from a sudden surge in oxygen availability or the activation of growth-promoting erythropoietin receptors within tumor tissues. In all hypoxia subtypes, despite a critical reduction in oxygen supply to tissues, factors such as the perfusion-dependent delivery of diagnostic and therapeutic agents, the supply of essential nutrients, the clearance of metabolic waste products, and the tissue’s capacity for repair can vary or remain unaffected [29]. Martijn and colleagues analyzed four clinical low-oxygen gene expression profiles and compared them to corresponding acute and chronic hypoxia profiles obtained from *in vitro* experiments. Their results showed that the acute hypoxia profile is a more robust prognostic indicator for patients with advanced-stage head and neck cancer than the chronic hypoxia profile [48]. Nonetheless, much of the existing research is confined to experimental and preclinical studies. Direct clinical evidence regarding both acute and chronic hypoxia remains limited, primarily due to the lack of reliable detection and quantification methods. Therefore, translating experimental findings into clinical practice requires the urgent adoption of advanced technologies, including improved imaging techniques and the use of validated modeling, before any changes to current radiotherapy regimens can be effectively implemented [41]. Recently, researchers have identified and classified hypoxia in specific tumors, such as hepatocellular carcinoma (HCC), into two distinct subtypes through detailed hypoxic transcriptome analyses. This nuanced classification has revealed unique clinical and pathological features characterizing each subtype. Moreover, observed heterogeneity in tumor-infiltrating immune cells highlights the complex and varied TMEs associated with the two distinct hypoxic subtypes [49].

### Determination of tumor hypoxic regions

Evidence suggests the presence of hypoxic regions within the internal milieu of many solid tumors [50]. Identifying these hypoxic areas is crucial for effective tumor treatment. Currently, two primary methods are used to detect tumor hypoxia: invasive polarographic oxygen electrode measurements and non-invasive medical imaging techniques. Polarographic Oxygen Electrodes. Although the concept of tumor hypoxia was proposed 65 years ago based on histological observations, it was not confirmed until direct measurements of oxygen concentration were conducted using polarographic oxygen electrodes in various tumor types [51, 52]. Results from these electrodes provide a visual representation of oxygen levels within tumors and are often regarded as the gold standard for hypoxia detection [53]. However, despite their effectiveness, polarographic oxygen electrodes have notable limitations. They require multiple measurements, making the process time-consuming and labor-intensive. Moreover, as the electrode moves through the stroma and fibrous tissues, it may experience pressure, potentially introducing biases into the data. Additionally, the invasive nature of the procedure—requiring electrode insertion into the tumor—can cause patient discomfort and even elevate the risk of tumor metastasis [54]. Therefore, there is a pressing need for newer, more versatile techniques to address these drawbacks.

### Medical imaging

Molecular imaging techniques, combined with appropriate contrast agents, enable the accurate detection of hypoxic tissues within tumors by overcoming the limitations of oxygen electrode measurements and providing comprehensive information on oxygen-deprived areas throughout the entire tumor. Given the close correlation between intratumoral hypoxia and factors, such as malignancy and drug resistance, non-invasive hypoxia imaging via molecular methods offers critical information for assessing tumor prognosis. This approach supports more effective clinical evaluations and ultimately enhances treatment success rates. Currently, several molecular imaging modalities are available for tumor hypoxia imaging, including positron emission tomography (PET). Among PET tracers, ^18^F-fluoromisonidazole (^18^F-FMISO) is the most widely used for detecting hypoxia in human studies [55]. In a study by Panek et al., 10 patients with locally advanced HNSCC underwent two 3T magnetic resonance imaging (MRI) scans at specific intervals prior to chemotherapy and radiotherapy. The findings showed that T2 measurements were reproducible and highly sensitive to changes in blood oxygen saturation, with significant differences detected under varying conditions. This confirms that T2-based 3T MRI can effectively monitor oxygenation changes in tumor tissues, highlighting the crucial role of MRI in detecting tumor hypoxic regions [56].

Additionally, optical imaging [57, 58] and photoacoustic imaging [59, 60] are also important techniques for tumor hypoxia imaging. Evidence further suggests that tumor vascular analysis, assessment of metabolic activity, evaluation of DNA damage in tumor cells, detection of radioresistant hypoxic markers, and identification of endogenous biological hypoxia markers can also serve as methods for detecting tumor hypoxia [61, 62]. Recent technological advancements have significantly improved real-time, high-resolution oxygen mapping [63], and the development of 3D microtumor “organoid” platforms [64]. These innovations offer distinct advantages for understanding the hypoxic state of tumors. Overall, detecting intratumoral hypoxia provides a foundation for assessing tumor prognosis and adopting targeted interventions. Hypoxia-targeted therapies can help overcome resistance encountered in traditional treatments, making them highly relevant for clinical strategies aimed at preventing and treating tumor hypoxia while enhancing the body’s adaptive capabilities. Moreover, gene expression profiling has been proposed as a means of detecting tumor hypoxia, with several hypoxia-specific gene expression profiles available [48]. However, this method can only determine the presence of hypoxia in the tumor and cannot precisely localize hypoxic regions.

Research into hypoxic conditions at the micro-regional scale of tumors has long attracted significant attention. Studies have shown that structural deficiencies in the tumor microvascular network lead to heterogeneous oxygen distribution. The network’s signal transduction capacity is impaired, and unlike normal microvessels, it cannot effectively regulate vessel diameter according to hemodynamic and metabolic needs. As a result, discrepancies in vessel diameters, uneven blood flow distribution, inadequate perfusion in certain areas, restricted oxygen supply, the formation of hypoxic regions, and heterogeneous oxygen distribution occur. This heterogeneity influences tumor growth and development, making tumor cells in hypoxic areas more resistant to chemotherapy and radiotherapy, thereby reducing treatment efficacy [65]. In addition, some researchers have introduced a data-driven mechanistic modeling approach, using patient-derived tumor xenograft models of three tumor types (breast cancer, ovarian cancer, and pancreatic cancer) as study subjects. Marker densities are extracted through image processing, and these data are then used to formulate reaction–diffusion equations that describe oxygen distribution. From these, a hypoxia distribution model is derived. Results show that due to the uneven distribution of blood vessels within individual tumors, oxygen supply varies considerably. Furthermore, the hypoxic characteristics differ significantly among tumor types [66]. A comprehensive comparative analysis of hypoxic variations across different clinical tumor types has also been conducted. As shown in Table 2, for multiple tumor types—including brain metastases, breast cancer, and cervical cancer—details, such as tumor stage, number of cases, intratumoral oxygen levels, and the proportion of areas with HF < 2.5 mmHg are presented, highlighting differences in hypoxic characteristics, intratumoral heterogeneity, and spatially variable hypoxia among tumor types.

**Table 2:** Analyzing the disparities in hypoxic zones across different clinical categories of tumors

**Tumor type**	**Stage**	**Number of cases**	**Oxygen level within tumor**	**HF < 2.5 mmH g**
Brain metastasis [67]	IV	5	Median: 3–23.7 mmHg	42.1%
Breast cancer [68]	Na	15	Median: 3–74 mmHg	5.8%
Cervical cancer [69]	Na	59	Mean: 10 mmHg	29.0%
Cervical cancer [70]	I–IV	18	Mean: 21.1 mmHg	Na
Glioblastoma [67]	Na	10	Median: 0.1–24.3 mmHg	26.0%
Head and neck cancer [71]	III, IV	10	13.9 ± 8.0 mmHg	8.3%
Head and neck cancer [72]	Na	16	25.6 ± 20.2 mmHg	Na
Head and neck cancer [73]	Na	133	Primary: 12 (0–58 mmHg) Metastases: 13 (0–50 mmHg)	Na
Metastatic melanoma [74]	IV	18	Mean: 11.6 mmHg	15.0%
Non-small cell lung cancer [75]	I–III	20	16.6 (0.7–46 mmHg)	Na
Pancreatic tumor [76]	Na	7	Median: 0–5.3 mmHg	59.0%
Prostate cancer [77]	I–III	59	2.4 (0–65 mmHg)	Na
Rectal carcinoma [78]	Na	15	25.7 ± 17.9 mmHg	Na
Renal cell carcinoma [79]	Na	3	13.3 ± 17.4 mmHg	Na
Vulvar cancer [80]	Na	Primary 15 Recurrent 19	Primary: 16.4 ± 14.4 mmHg Recurrent: 15.7 ± 13.4 mmHg	Na

### Tumor hypoxia signaling pathway

The HIF signaling pathway plays a central role in the response of tumor cells to hypoxic environments. It significantly influences various physiological and pathological processes, including angiogenesis, metabolism, and tumor development [81, 82]. Numerous studies have explored the mechanisms associated with this pathway. Some have shown that the small molecule inhibitor SU5416 can markedly downregulate the expression levels of VEGF and HIF-1α in ovarian cancer cells by inhibiting the PI3K/Akt signaling pathway. Conversely, 4-hydroxyestradiol (4-OHE2) has been found to promote the high expression of HIF-1α through regulation of the same pathway, underscoring the critical role of PI3K/Akt in modulating HIF-1α expression [83, 84]. Beyond the PI3K/Akt pathway, HIF signaling is also interconnected with other molecular pathways. For instance, studies on liver injury following traumatic hemorrhage have highlighted a strong association between the MAPK pathway and HIF activity. Traumatic hemorrhage significantly reduces liver p38 MAPK activity while simultaneously inducing a marked increase in HIF-1α expression. Treatment with the p38 MAPK inhibitor SB203580 inhibits the restoration of p38 MAPK activity and prevents the reduction of HIF-1α expression, confirming the regulatory role of the MAPK pathway in HIF-1α expression [85]. In addition, HIF signaling can be modulated by several other pathways, including the JAK-STAT3 pathway [86], the NF-κB pathway [87], the Notch pathway [88], and the Wnt/β-catenin pathway [89].

### Hypoxia and genetic damage repair

Hypoxia is a common feature of many solid tumors and is known to induce various forms of genetic damage. Under low oxygen conditions, cancer cells often develop increased resistance to treatments, such as radiotherapy and chemotherapy. This resistance is attributed to changes in the frequency of DNA lesions, including single- and double-strand breaks (DNA-SSBs and DNA-DSBs), DNA–DNA cross-links, base damage, and DNA–protein cross-links [90]. Studies using both *in vitro* and *in vivo* models of hypoxic cancers have reported gene mutation rates two to five times higher than those observed under normoxic conditions [91–93]. Moreover, tumor cells *in vivo* exhibit more extensive genomic rearrangements and a higher prevalence of point mutations and small deletions in specific genes compared to cells grown *in vitro*. *In vitro* experiments have also shown that hypoxic stress can trigger excessive DNA replication, leading to gene amplification and the emergence of genetically unstable regions. Particularly concerning is evidence from mouse models indicating that hypoxia in fibrosarcoma and melanoma cells not only promotes genomic instability but also enhances metastatic potential [94]. These mutations and chromosomal breaks contribute to oncogene activation, facilitating the emergence of cancer cell variants with greater proliferative capacity [95]. However, some studies report that exposing cancer cell lines to physiological levels of chronic hypoxia (0.2% oxygen) can cause the accumulation of γH2AX in a HIF-dependent manner, without detectable DNA strand breaks [96]. Alfredo’s research has shown that severe hypoxia and acidosis in breast cancer cells can induce the expression of lncMat2B, a long non-coding RNA (lncRNA) found in hypoxic tumor-initiating cells (TICs) within multicellular tumor spheroids (MCTS). Overexpression of lncMat2B enhances resistance to cisplatin by reducing drug-induced DNA damage and promoting DNA repair [97]. Notably, even moderate hypoxia can induce replication stress and activate proteins involved in the DNA damage response (DDR) pathway [98]. The specific nature of hypoxia—acute vs chronic—also influences the DNA repair mechanisms involved in cancer progression. Acute hypoxic stress can rapidly alter DNA repair pathways through post-translational modifications, while chronic hypoxia tends to downregulate DNA repair proteins at the transcriptional and/or translational levels. Prolonged moderate hypoxia may further contribute to epigenetic regulation of DNA repair genes, adding to the complexity of cellular responses under hypoxic conditions [94]. In conclusion, hypoxia induces both genetic damage and altered DNA repair in cancer cells, promoting tumor cell proliferation and posing a multifaceted challenge that requires further investigation.

### Hypoxia-driven metabolic reprogramming

The metabolic reprogramming of tumor cells in response to hypoxia is a critical area of research, as it not only influences tumor growth and survival but also plays a key role in developing effective therapeutic strategies. Among the various mechanisms involved in hypoxia-induced metabolic changes, HIF-1α plays a central role. Activated under low-oxygen conditions, HIF-1α orchestrates a wide range of metabolic adaptations within cancer cells. For example, Faubert et al. [99] demonstrated that loss of the tumor suppressor LKB1 promotes metabolic reprogramming through HIF-1α, underscoring its importance in cancer metabolism. Similarly, Chen et al. [100] showed that miR-3662 suppresses HCC growth by inhibiting the HIF-1α-mediated Warburg effect—a hallmark of cancer metabolism characterized by elevated glycolysis despite the presence of oxygen. These findings highlight the diverse molecular mechanisms by which HIF-1α contributes to cancer metabolic regulation. In addition to cancer cells themselves, metabolic reprogramming in CAFs significantly influences tumor–stroma interactions. Fiaschi et al. [101] reported that CAFs promote tumor progression by enhancing EMT, supporting CSC traits, and facilitating metastasis. CAFs can also adopt Warburg metabolism, which alters their own metabolic state and impacts the metabolism of neighboring cancer cells. This reciprocal reprogramming emphasizes the critical role of the TME in shaping cancer metabolism. Beyond HIF-1α, sirtuins—NAD^+^-dependent deacetylases—also regulate metabolic reprogramming under hypoxia. Notably, SIRT6, a multifunctional member of the sirtuin family, inhibits the transcriptional activity of MYC and HIF-1α—two key transcription factors involved in promoting glycolysis, proliferation, and biomass production. Through deacetylation, SIRT6 modulates the activities of both MYC and HIF-1α, forming a complex regulatory network. The dynamic balance among these factors profoundly influences the direction and intensity of tumor metabolism and the broader biological behavior of tumors [102]. More recently, research has identified glycogen branching enzyme 1 (GBE1) as a downstream target of HIF-1α in lung adenocarcinoma. This study established a link between GBE1 expression and metabolic alterations that drive tumor progression, illustrating how specific metabolic enzymes mediate hypoxia-induced changes in tumor behavior [103]. In conclusion, tumor metabolic reprogramming in response to hypoxia is a multifaceted process involving numerous signaling pathways, metabolic regulators, and microenvironmental interactions. HIF-1α emerges as a central orchestrator of these adaptations, exerting a profound impact on cancer cell metabolism.

### Hypoxia promotes tumor progression

The hypoxic microenvironment within tumor tissues—marked by low oxygen levels—and the hypoxic tumor cells within these regions play a critical role in driving tumor progression [104]. Numerous studies have confirmed that such conditions promote tumor growth by activating molecular pathways and signaling cascades, ultimately contributing to cancer aggressiveness and treatment resistance.

### Hypoxia promotes tumor angiogenesis

One of the critical roles of the hypoxic microenvironment in tumor proliferation is its promotion of new blood vessel formation, a process that supplies essential nutrients for tumor growth. This angiogenic process involves the HIF family. Among the known HIF subtypes, hypoxia-inducible factor 1 (HIF-1) is particularly associated with angiogenesis [105, 106]. Under low-oxygen or hypoxic conditions, HIF-1 plays a central role in orchestrating the tumor cell response. It exerts its effects by directly targeting these cells and inducing the production of vascular endothelial growth factor (VEGF), a potent signaling protein. VEGF’s action is multifaceted: it not only promotes the proliferation of endothelial cells, which line blood vessels, but also increases vascular permeability. This dual effect triggers a cascade that facilitates the formation of new blood vessels in and around the TME. By enhancing vascular permeability, VEGF allows for more efficient transport of nutrients to tumor cells, thereby supporting their aggressive growth and survival. Tumor cells require abundant resources to sustain their rapid proliferation. Additionally, studies have shown that HIF can activate carbonic anhydrase-9 (CA9), which also plays a vital role in hypoxia-induced angiogenesis [107]. In essence, HIF activation under hypoxic conditions establishes a proangiogenic environment that strengthens the tumor’s ability to thrive by ensuring a robust and continuous nutrient supply through an expanded vascular network [108, 109]. EVs, commonly present in the TME, are small, membrane-bound vesicles secreted by various cell types and play essential roles in intercellular communication. Under hypoxic conditions, tumor cells exhibit accelerated proliferation and heightened metabolism, leading to a marked increase in the secretion of EVs [110, 111]. These vesicles, rich in nucleic acids, serve as critical carriers of molecular signals that drive tumor cell proliferation [112]. Notably, recent studies have highlighted the role of miR-23a, a microRNA packaged within exosomes released by tumor cells under hypoxic stress. Research indicates that oxygen deprivation significantly increases miR-23a levels in these vesicles. Further investigation has revealed that miR-23a exerts its tumor-promoting effects by targeting and suppressing SIRT1, a protein essential for normal cellular function. By silencing SIRT1, miR-23a enhances the proliferation and recruitment of endothelial cells, the primary builders of blood vessels. This manipulation of endothelial cell behavior is key to angiogenesis, a hallmark of tumor growth and progression [113]. Moreover, the increased presence of EVs in the hypoxic microenvironment, particularly those carrying peroxidase enzymes, is also linked to the initiation of neovascularization in tumor tissues [114].

### Hypoxia promotes tumor cell invasion

**Figure 2.**
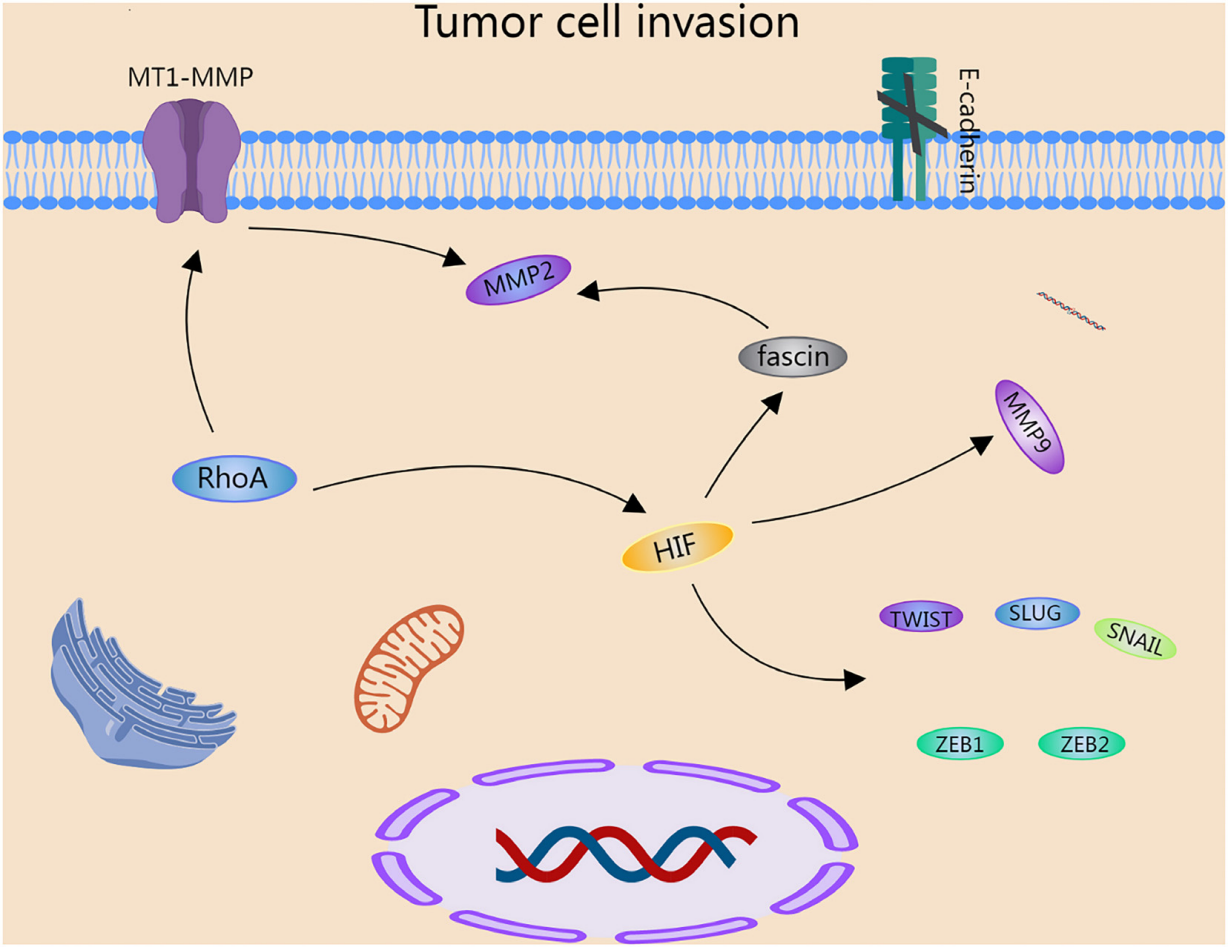
**Hypoxia promotes tumor cell invasion.** In the hypoxic environment, tumor cells produce RhoA, which leads to the upregulation of the cell surface matrix metalloproteinase MT1-MMP. MT1-MMP activates another matrix metalloproteinase, MMP-2, triggering EMT in tumor cells. Furthermore, RhoA regulates the expression level of HIF, subsequently increasing the expression of downstream factor fascin and thus enhancing the expression of MMP-2. High expression of HIF can promote the expression of molecules, such as TWIST, SLUG, SNAIL, ZEB1, and ZEB2, which act to reduce the expression of E-cadherin on the cell surface, ultimately facilitating EMT and promoting tumor cell invasion. HIF: Hypoxia-inducible factor; EMT: Epithelial-to-mesenchymal transition.

In a low-oxygen environment, the process begins with the activation of the small GTPase RhoA in breast cancer cells—an essential step that initiates a cascade of downstream signaling pathways. This signaling ultimately increases the expression of the cell surface metalloproteinase MT1-MMP, a key enzyme responsible for degrading components of the ECM. By disrupting ECM integrity, MT1-MMP facilitates tumor cell migration and invasion. Under hypoxic conditions, MT1-MMP expression not only increases but also contributes to the activation of another matrix metalloproteinase, MMP-2 [115]. Simultaneously, activation of the small GTPase Ras plays a critical role in stabilizing HIF-1α, a transcription factor that promotes the expression of MMP-9 [116] and fascin—a protein that bundles actin filaments and enhances cancer cell migration, dispersion, and invasion. Fascin further regulates MMP-2 expression through the coordinated action of protein kinase C (PKC) and extracellular signal-regulated kinase (ERK) [117]. Together, the upregulation and activation of MMP-2 and MMP-9 synergistically increase cancer cell invasiveness, enabling them to penetrate and spread throughout the body. A key event in cancer invasion is EMT, during which cells shift from a stationary epithelial phenotype—characterized by E-cadherin expression, a rigid cytoskeleton, and strong cell-cell adhesion—to a mobile mesenchymal phenotype, marked by vimentin expression [118]. In various cancer subtypes, EMT is driven by a group of transcriptional repressors, including TWIST, SLUG, SNAIL, ZEB1, and ZEB2, which suppress epithelial gene expression such as that of E-cadherin. Notably, at least one of these repressors is regulated by HIF, a central mediator of the tumor hypoxia response [119]. Emerging evidence suggests that hypoxia contributes to the aberrant regulation of E-cadherin expression, ultimately reducing its protein levels. This decline compromises cell–cell cohesion and promotes cancer cell invasion and metastasis [120–122]. Collectively, these findings indicate that hypoxia supports tumor cell invasion by inducing EMT. In summary, hypoxia activates RhoA, which elevates MT1-MMP levels and promotes HIF signaling. MT1-MMP activates MMP-2, while HIF upregulates fascin, which further enhances MMP-2 expression. HIF also induces EMT by upregulating transcriptional repressors like TWIST, SLUG, SNAIL, ZEB1, and ZEB2, leading to E-cadherin downregulation. These events together promote tumor invasion and metastasis (Figure 2).

### Hypoxia promotes tumor immune escape

**Figure 3.**
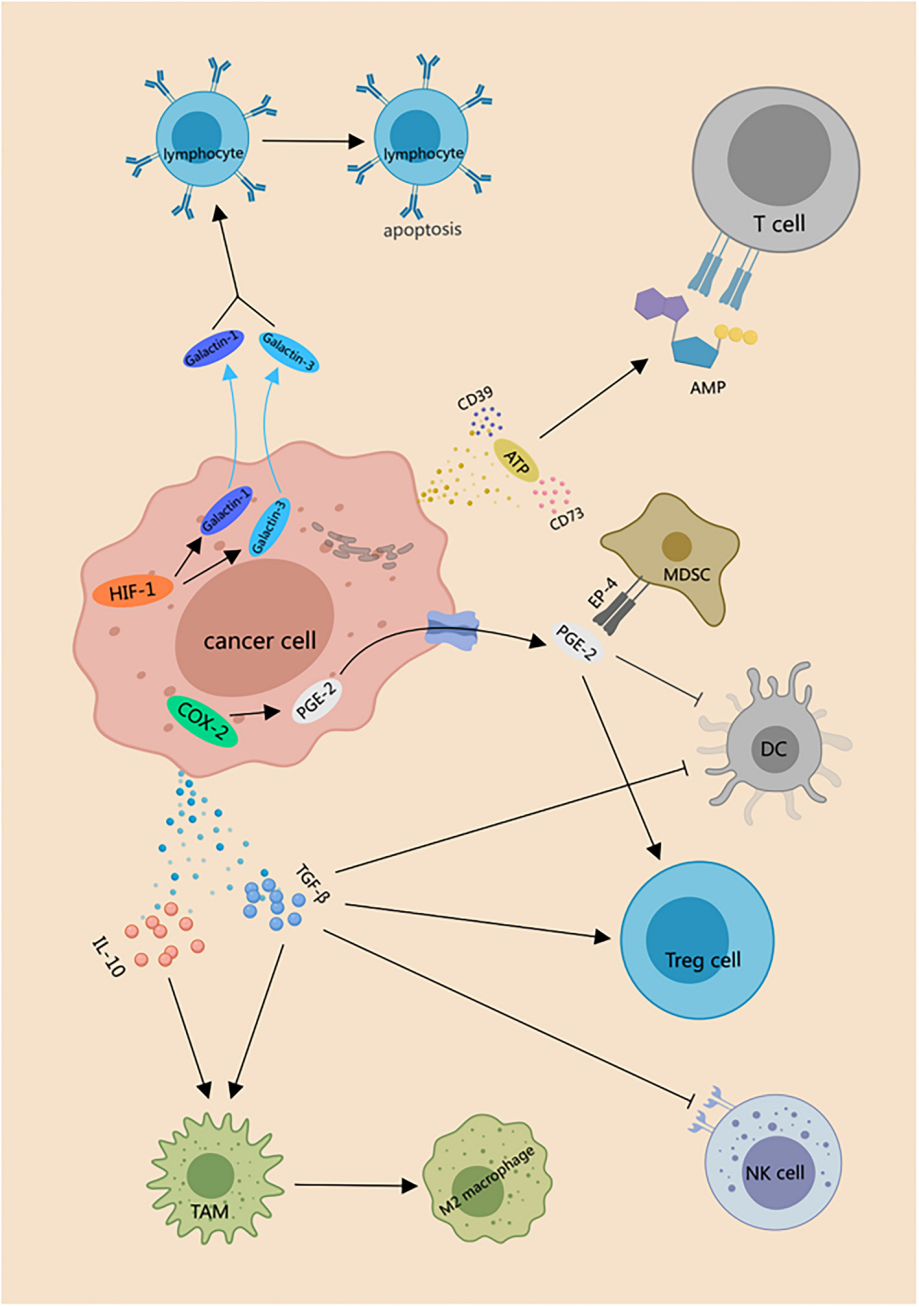
**Hypoxia induces the release of immune suppressive molecules from tumor cells.** Dying cancer cells release ATP, and CD73 and CD39 metabolize it into adenosine, which binds to specific receptors on T cells, elevating intracellular cAMP levels, thereby inhibiting T cell function. Under hypoxic conditions, tumor cells also release IL-10 and TGF-β, leading to the differentiation of TAMs into M2 macrophages with immune-suppressive activity. TGF-β inhibits T cell proliferation and effector functions, promotes the generation of Tregs, and simultaneously blocks the receptor expression necessary for NK cells to exert cytotoxic functions. TGF-β also downregulates the expression of CD1d, an antigen-presenting surface molecule on DCs, thereby inhibiting T cell differentiation and function. Hypoxia induces the activation of HIF-1, resulting in the production of galectin-1 and galectin-3, leading to apoptosis of activated lymphocytes. Hypoxic tumor cells, through upregulation of COX-2, increase the expression of PGE2, inhibiting DC maturation, promoting Treg differentiation, and triggering immune suppression. Additionally, PGE2 can bind to the EP-4 receptor on MDSCs, exerting immune-suppressive effects. NK: Natural killer; MDSC: Myeloid-derived suppressor cell; TAM: Tumor-associated macrophage; DC: Dendritic cell; COX-2: Cyclooxygenase-2; PGE2: Prostaglandin E2; HIF-1: Hypoxia-inducible factor 1.

Hypoxia induces the release of immunosuppressive molecules from tumor cells. Under severe hypoxic conditions, dying cells release ATP, which is subsequently metabolized into adenosine by the enzymes CD73 and CD39 [123]. Extracellular adenosine binds to specific receptors on the surface of T cells, leading to an increase in intracellular cAMP levels. This biochemical shift exerts an inhibitory effect on T cell function [124]. By exploiting this immunosuppressive pathway, tumors evade immune detection, facilitating unchecked growth and metastasis. In hypoxic environments, tumor cells also secrete immunosuppressive cytokines, such as IL-10 and TGF-β, which play pivotal roles in shaping the TME. These cytokines drive the polarization of TAMs into the M2 phenotype, characterized by immunosuppressive activity. M2 macrophages suppress anti-tumor responses by inhibiting T cell proliferation and effector functions [125]. Moreover, TGF-β enhances immune evasion by promoting the expansion of regulatory T cells (Tregs), which suppress immune responses against tumor cells. TGF-β also diminishes the cytolytic activity of natural killer (NK) cells by downregulating their activating receptors, impairing their ability to identify and kill tumor cells [126]. Additionally, TGF-β reduces the expression of the antigen-presenting molecule CD1d on dendritic cells (DCs), limiting their capacity to present tumor antigens and initiate effective T cell responses [127]. Studies have shown that in hypoxic colon and HNSCCs, HIF-1 plays a central role in orchestrating an immunosuppressive network. HIF-1 promotes the expression of galectin-1 and galectin-3, which induce apoptosis in activated lymphocytes [128]. This immunosuppressive effect is compounded by tumor-mediated upregulation of cyclooxygenase-2 (COX-2), which catalyzes the production of prostaglandin E2 (PGE2). Elevated PGE2 levels further increase adenosine/cAMP signaling in effector T cells [129] and activate the EP-4 receptor on myeloid-derived suppressor cells (MDSCs), enhancing their immunosuppressive function [130]. These mechanisms collectively inhibit DC maturation and promote the differentiation of (Tregs), resulting in widespread immune suppression that favors tumor progression. Thus, within a hypoxic TME, cancer cells deploy a multifaceted immune evasion strategy driven by a network of molecular signals and pathways. A central component of this strategy is the accumulation of adenosine, which impairs T cell-mediated anti-tumor responses. In tandem, the secretion of immunosuppressive cytokines, such as IL-10 and TGF-β further compromises immune surveillance, fostering a tumor-supportive microenvironment that enables ongoing growth and metastasis (Figure 3). Hypoxia also alters the expression of immune checkpoint regulators on tumor cells. Beyond impairing the cytolytic capacity of immune effector cells, hypoxia reshapes the immunological profile of tumor cells to make them less detectable by the immune system. One such mechanism involves HIF-1α, which enhances the enzymatic activity of ADAM10, a metalloproteinase that cleaves membrane-bound MHC class I chain-related molecule A (MICA). This cleavage results in the release of soluble MICA into the extracellular space [131]. Soluble MICA has reduced capacity to engage the NKG2D receptor on NK and T cells, thereby weakening their ability to target and eliminate malignant cells [132]. In addition, hypoxic stress stabilizes and promotes the accumulation of HIF-1α, which upregulates the expression of programmed death-ligand 1 (PD-L1). PD-L1 binds to the PD-1 receptor on cytotoxic T lymphocytes (CTLs), delivering an inhibitory signal that induces T cell exhaustion or apoptosis [133]. This interaction diminishes CTL cytolytic activity, allowing tumor cells to evade destruction by the immune system [134].

**Figure 4.**
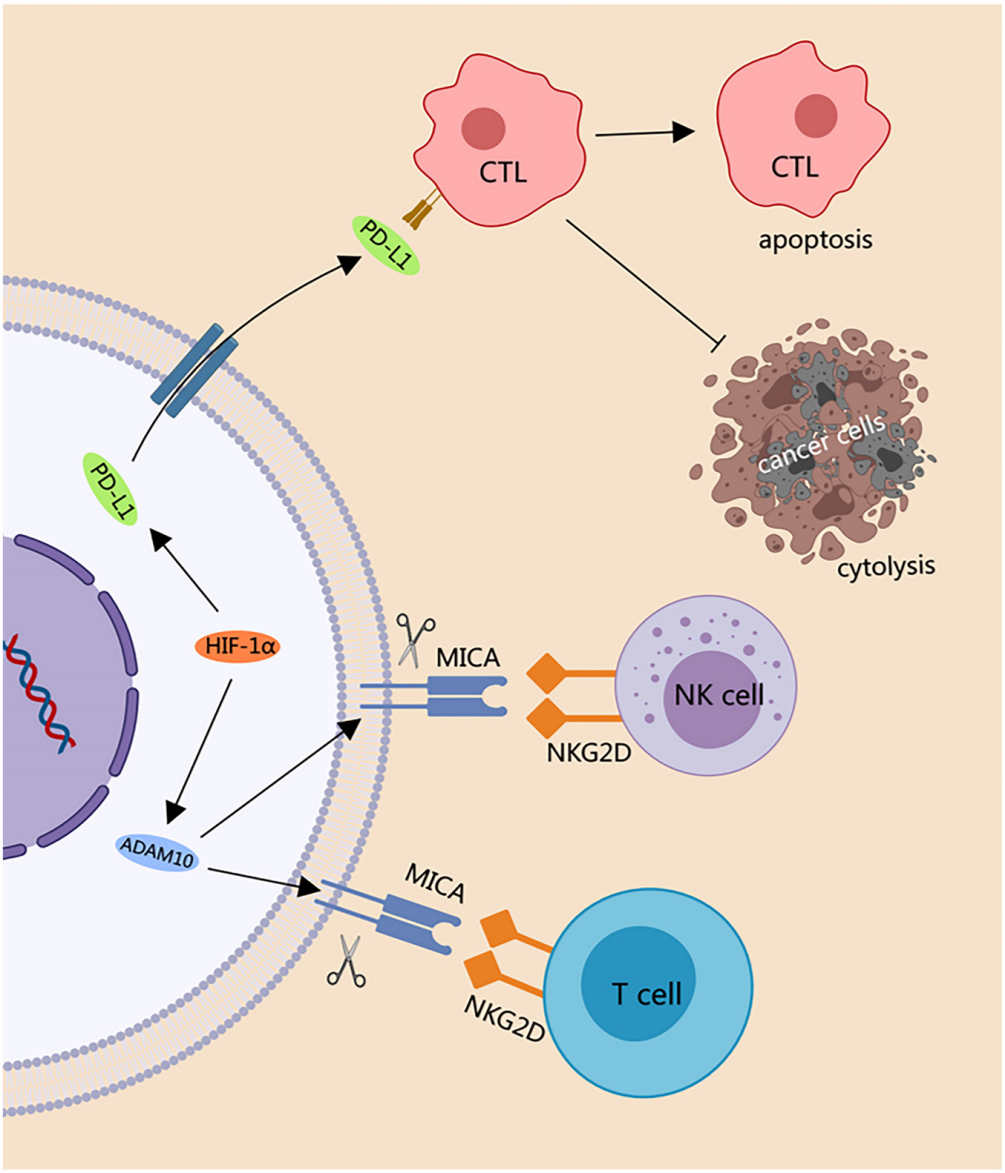
**Hypoxia-induced changes in tumor cell checkpoint regulator expression.** HIF-1α activates the expression of metalloproteinase ADAM10, leading to the shedding of cell surface pressure-induced MICA. This, in turn, reduces the binding with the receptor NKG2D on NK cells and T cells. Hypoxia-induced activation of HIF-1α in tumor cells also results in high expression of PD-L1. The binding of PD-L1 with the receptor PD-1 on CTL increases the apoptosis of CTL, simultaneously resisting CTL-mediated lysis of tumor cells. MICA: MHC class I chain-related molecule A; PD-L1: Programmed death-ligand 1; CTL: Cytotoxic T lymphocyte; NK: Natural killer; HIF-1α: Hypoxia-inducible factor-1α.

These dual actions of hypoxia—through the induction of ADAM10 activity leading to MICA shedding and through the upregulation of PD-L1 on the tumor cell surface—underscore the multifaceted mechanisms by which tumors can evade the immune system (Figure 4). Studies have shown that 32-134D, a low-molecular-weight compound that inhibits HIF-1/2-mediated gene expression in HCC cells, when combined with anti-PD-1 therapy, increases HCC eradication rates in mice from 25% to 67% [135]. Such findings point to promising therapeutic directions. Given the significance of these discoveries, understanding how hypoxia affects immune checkpoints is essential for developing targeted therapies to counteract tumor immune evasion. The immune effects of hypoxia-induced angiogenesis growing evidence suggests that hypoxia-induced angiogenesis also contributes to immune tolerance. Hypoxia triggers a wide array of physiological responses, most notably the upregulation of VEGF, a key factor in angiogenesis [108, 109]. VEGF has been shown to obstruct the tumor-induced differentiation of DCs. *In vitro* experiments demonstrate that VEGF-specific neutralizing antibodies can counteract the suppressive effects of tumor cell-derived media on the differentiation of DCs from hematopoietic progenitor cells. *In vivo* studies corroborate these findings: administration of recombinant VEGF to tumor-free mice inhibits DC development and increases the production of GR1^+^ immature myeloid cells (iMCs). Conversely, treatment with VEGF-neutralizing antibodies in tumor-bearing mice enhances DC differentiation and increases the number of mature DCs. These results suggest that VEGF impairs DC maturation and, consequently, their ability to present tumor-associated antigens to helper T cells [136]. Additionally, VEGF promotes the accumulation of MDSCs in tumors and secondary lymphoid tissues, suppressing anti-tumor T cell responses. It also stimulates the release of factors that facilitate angiogenesis and metastasis, thereby promoting tumor progression [137]. Clinical studies have investigated the effects of VEGF on DCs and iMCs in cancer patients. For instance, Osada et al. [138] studied 41 cancer patients (with lung, breast, colorectal, or unknown primary cancers) alongside 30 healthy controls. They found that DCs in cancer patients were less mature and skewed toward an immunoregulatory DC2 phenotype, accompanied by increased iMC numbers. A positive correlation was observed between VEGF levels and both DC2 and iMC counts, suggesting VEGF contributes to immune dysfunction by inhibiting DC maturation. Treatment with anti-VEGF antibodies, such as bevacizumab, led to decreased iMC counts and increased DC numbers in some patients, along with improved immune markers like IL-12 secretion and enhanced antigen presentation. These findings highlight VEGF’s role in DC dysfunction and tumor immune suppression and suggest that anti-VEGF therapy could improve immune responses in cancer patients. The direct impact of hypoxia on immune effector cells also directly affects immune effector cells, particularly T cells and natural killer (NK) cells. First, hypoxia can suppress T cell functionality—key players in the immune response—through several mechanisms. Research by Clambey et al. [139] has shown that low-oxygen environments increase the expression of the FoxP3 gene in T cells, leading to a rise in Tregs and a concurrent inhibition of effector T cell proliferation. HIF-1α is central to this process, stabilizing FoxP3 expression and influencing its function. Furthermore, hypoxia can inhibit the expression of antigen-presentation molecules, such as MHC-II, CD80, and CD86 on DCs, which in turn diminishes effector T cell activation. These findings indicate that hypoxia can modulate T cell activity by altering immune effectors on their surface, potentially dampening anti-tumor responses [140]. However, contrasting data also exist. For example, Palazón et al. [141] demonstrated that HIF-1α can enhance the transcription of CD137—a key receptor in the TNF superfamily important for T cell activation. Their work revealed that the absence of HIF-1α leads to significantly reduced CD137 expression and impaired T cell activation. Collectively, these studies suggest that hypoxia may have a dual regulatory role in T cell function, necessitating further investigation into its complex immunomodulatory effects. Second, hypoxia severely impairs NK cell function, which is critical in defending against malignancies such as multiple myeloma. Oxygen deprivation reduces the surface expression of essential receptors like NKG2D and CD16 on NK cells, compromising their ability to recognize and targetabnormal cells. Additionally, hypoxia lowers intracellular levels of perforin and granzyme B—proteins vital for NK cell-mediated cytotoxicity. This reduced expression limits the ability of NK cells to kill multiple myeloma cells effectively [142]. These findings underscore the significant impact of hypoxia on immune effector cells and highlight the need to develop strategies to mitigate its immunosuppressive effects within the TME.

### Hypoxia promotes proliferation of CSC

Research indicates that various solid cancers are driven by a rare type of cell known as CSCs. Although CSCs make up only a small fraction of the total tumor, they possess the remarkable ability to regenerate and differentiate into diverse cancer cell types, giving them a significant advantage in spreading and resisting treatments. This suggests that CSCs are key players in both the formation and growth of tumors [143, 144]. Hypoxia has been identified as a critical factor that promotes the expansion of CSCs and enhances their tumorigenic potential across multiple cancer types. In glioblastoma, an especially aggressive brain cancer characterized by vigorous angiogenesis, hypoxia-inducible factors (HIFs) have been shown to regulate the tumorigenicity of glioma stem cells (GSCs). These GSCs, highly dependent on HIFs for survival, regeneration, and tumor contribution, are strategically located within specific tumor niches—such as perivascular regions and necrotic zones—where they can optimally exploit low-oxygen conditions [145, 146]. Similarly, in HCC, hypoxia accelerates disease progression by rewiring the metabolism of mesenchymal stem cells (MSCs) toward increased lipogenesis, thereby promoting tumor growth and enhancing malignancy [147]. Regarding the mechanisms by which hypoxia fosters CSC proliferation, research suggests that several key pathways are involved. Hypoxic conditions first induce the upregulation of HIF-1α, which subsequently activates the Notch 1 signaling pathway. Notch 1 is crucial for maintaining CSC stemness by regulating the balance between self-renewal and differentiation, primarily through interactions between its ligands and specific cell surface receptors [148]. Additionally, hypoxia can lead to the overexpression of HIF-2α, which activates the downstream gene Oct-4. Oct-4 functions as a barrier to cellular differentiation, further preserving the stem-like characteristics of CSCs within tumors [149, 150]. Collectively, these findings illuminate the complex interplay between hypoxia, CSCs, and the TME in driving tumor progression.

### Hypoxia and therapy resistance

Tumor hypoxia profoundly influences multiple treatment modalities, including chemotherapy, radiotherapy, and immunotherapy, through complex and intertwined mechanisms. Antiangiogenic therapies, designed to cut off the tumor’s blood supply, present a double-edged sword. While these therapies can initially suppress tumor growth, reports have shown that they often lead to increased hypoxia within the tumor. This heightened hypoxia subsequently drives the selection of more aggressive cancer phenotypes, ultimately correlating with poor patient prognosis. This duality underscores hypoxia’s role as both a potential therapeutic target and a formidable resistance mechanism [151]. In the domains of chemotherapy and radiotherapy, hypoxia—often exacerbated by anemia—greatly weakens the effectiveness of these treatments. Hypoxia may reduce the sensitivity of tumors to radiation and chemotherapy through one or several indirect mechanisms, including proteomic and genomic changes. These effects, in turn, can lead to increased invasiveness and metastatic potential, loss of apoptosis, and disrupted angiogenesis, further enhancing treatment resistance [152]. The role of HIF in mediating treatment resistance has been a focal point of extensive investigation. Under hypoxic conditions, research has shown that the expression of both DNA-dependent protein kinase (DNA-PK) and HIF-1α increases. Moreover, DNA-PK can directly interact with HIF-1. This regulatory relationship between DNA-PK and HIF-1 underpins the therapeutic resistance of hypoxic tumor cells, providing a new basis for strategies aimed at improving treatment efficacy [153]. Furthermore, complex relationships have been identified between HIF and various cell death pathways, such as autophagy. Hypoxia has been shown to trigger autophagy—a cellular survival mechanism that enables tumor cells to endure the stress imposed by therapeutic agents. This phenomenon is particularly evident in studies on glioblastoma, where hypoxia-induced autophagy promotes tumor cell survival, adding another layer of complexity to the treatment landscape [154]. The role of hypoxia in immunotherapy resistance has also been under scrutiny. Research has explored the correlation between tumor hypoxia and resistance to PD-1 blockade in HNSCC. Under hypoxic conditions, tumor cells undergo metabolic alterations. For instance, in a murine head and neck cancer model, anti-PD-1-resistant cell lines exhibit heightened oxidative metabolism, intensifying intratumoral hypoxia. Concurrently, this hypoxic state impacts immune cell infiltration, specifically reducing the infiltration of crucial CD8+ T cells and thereby weakening the immune system’s ability to eliminate tumor cells. Additionally, tumor hypoxia fosters the establishment of an immunosuppressive microenvironment. This includes modulating the expression of immunosuppressive molecules, inducing changes in the TME, and reshaping the functional distribution of immunosuppressive cells. These adaptations enable tumor cells to evade immune surveillance and cytotoxicity, ultimately resulting in resistance to immunotherapy [155]. Given these complex interactions, further research has identified HIF-1 as a key mediator of resistance to anti-PD-(L)1 therapies [156]. Currently, the development of hypoxia-targeted therapies faces significant challenges, and clinical trial outcomes have been inconsistent. Consequently, there is a growing consensus that a personalized approach is essential [157]. Nevertheless, there is cause for optimism, as innovative strategies to overcome hypoxia-induced resistance are emerging. One promising approach involves using pH-responsive liposomes to enhance sonodynamic therapy by improving oxygen delivery to hypoxic tumors [158]. In summary, tumor hypoxia represents a multifaceted and complex challenge that significantly contributes to treatment resistance across a spectrum of cancer therapies. A comprehensive understanding of the underlying molecular, cellular, and microenvironmental mechanisms is essential for developing innovative and personalized treatment strategies.

**Table 3:** Clinical trials and hypoxia-targeting drugs in tumor therapy

**Clinical trial ID**	**Tumor type**	**Primary outcome measures**	**Phase**	**Status**	**Target**	**Drug**	**Intervention**
NCT04648033	Non-small cell lung cancer	MTD	I	Completed	Hypoxia regions	Atovaquone	Oral
NCT00495144	Unspecified	MTD, DLTs	I	Completed	Hypoxia regions	TH-302	Intravenous infusion
NCT01497444	Kidney cancer or liver cancer	DLTs, MTD, RR	I/II	Completed	Hypoxia regions	TH-302	Intravenous infusion
NCT01149915	Advanced leukemias	MTD, DLTs, adverse events	I	Completed	Hypoxia regions	TH-302	Intravenous infusion
NCT00743379	Pancreatic cancer, prostate cancer, lung cancer	MTD, DLTs	I/II	Completed	Hypoxia regions	TH-302	Intravenous infusion
NCT01522872	Myeloma	Adverse events, MTD, DLTs, recommended doses	I/II	Unknown	Hypoxia regions	TH-302	Intravenous infusion
NCT01746979	Pancreatic cancer	Overall survival	III	Completed	Hypoxia regions	TH-302	Intravenous infusion
NCT01440088	Soft tissue sarcoma	Overall survival	III	Completed	Hypoxia regions	TH-302	Intravenous infusion
NCT02528526	Hepato-pancreato-biliary neoplasm	Safety and tolerability	Ib/IIa	Unknown	Hypoxia regions	OXY111A	Intravenous infusion
NCT05281003	Esophageal squamous cell carcinoma	RR, major hypoxia signals	II	Recruiting	PD-1	Pembrolizumab	Intravenous infusion
NCT04114136	Unspecified	RR	II	Recruiting	PD-1	Nivolumab or pembrozilumab	Intravenous infusion
NCT01763931	Breast cancer	Change in HIF-1α expression	II	Completed	HIF-1α	Digoxin	Oral
NCT04954599	Unspecified	Adverse events, biochemical test abnormalities	I/IIa	Recruiting	Hypoxia regions	CP-506	Intravenous infusion
NCT05119335	Kidney cancer	DLTs, recommended doses for expansion, RR, recommended phase 2 dose	I/II	Recruiting	HIF-2α	NKT2152	Oral
NCT03098160	Unspecified	Proper dose	I	Unknown	Hypoxia regions	Evofosfamide	Intravenous infusion
NCT02564614	Hepatocellular carcinoma	Change in HIF-1α expression	I	Completed	HIF-1α	RO7070179	Intravenous infusion
NCT00090727	Unspecified	Na	I	Unknown	Hypoxia regions	AQ4N	Intravenous infusion
NCT00466583	Lymphoma	MTD	I	Completed	HIF-1α	EZN-2968	Intravenous infusion
NCT00886405	Non-small cell lung cancer	Time to progression, over-all survival	II	Unknown	HIF-1α	Nitroglycerin	Skin adherence
NCT01950689	Head and neck squamous cell carcinoma	Locoregional control	III	Completed	Hypoxia regions	Nimorazole	Oral

HIF-1α: Hypoxia-inducible factor-1α.

### Hypoxia and cancer prognosis

Tumor hypoxia plays a pivotal role in cancer prognosis and therapeutic outcomes. Extensive research has explored the relationship between hypoxia levels and disease progression across various cancer types. While some clinical studies suggest that tumor hypoxemia may have no significant impact on patient survival [159]—potentially influenced by dynamic changes within the TME—the majority of evidence supports the view that hypoxemia contributes to poorer prognostic outcomes. As early as 2003, Koukourakis et al. [160] analyzed 76 cases of non-small-cell lung cancer (NSCLC), examining the correlation between lactate dehydrogenase-5 (LDH-5) and HIF expression and patient prognosis. By comparing patients with varying levels of LDH-5 and HIF expression, they assessed the impact of these biomarkers on survival. Their research found that elevated LDH-5 expression, particularly when combined with high levels of HIF1α and HIF2α, was associated with worse survival outcomes. Given that LDH-5 and HIFs are key molecules involved in tumor hypoxia, this suggests that the hypoxic state of tumor cells is a significant biological factor influencing prognosis in NSCLC. Similar observations have been made in gastric adenocarcinoma, where upregulation of HIF-1α is also associated with an unfavorable prognosis [161, 162]. In colorectal cancer, hypoxia profoundly affects the behavior of immune cells within the TME, influencing the activity and polarization of T cells, macrophages, and tumor-infiltrating lymphocytes. Hypoxia upregulates the expression of chemokines, such as CCL2, CCL4, CCL5, and CSF1, promoting the recruitment of immunosuppressive M2 macrophages. This facilitates tumor immune escape and suppresses anti-tumor immune responses, thereby significantly impacting disease progression, treatment response, and overall patient prognosis [163]. Additionally, research has identified hypoxia-related lncRNAs as potential biomarkers for predicting breast cancer prognosis and informing future therapeutic strategies [164, 165]. However, contradictory findings also highlight the complex relationship between tumor hypoxia and patient outcomes. For instance, Tribbles homolog 3 (TRIB3)—a cytokine induced by hypoxia and involved in various cell survival pathways—shows elevated mRNA levels associated with poor prognosis in breast cancer patients, yet paradoxically, high protein expression of TRIB3 is linked to better prognosis. This underscores the intricate interplay between TRIB3 expression and cancer progression, further reflecting the complexity of hypoxia’s impact on prognosis [166, 167]. Different HIF isoforms also play distinct roles in tumor biology. In xenograft models, HIF2α, rather than HIF1α, has been shown to drive tumor growth. Similarly, in animal models, activation of HIF2α or replacement of HIF1α with HIF2α promotes aggressive tumor growth and invasion, whereas overexpression of stable HIF1α inhibits tumor growth. Despite these insights into their roles within cancer cells, the functions of HIF1α and HIF2α in the tumor stroma remain largely unexplored [168]. Moreover, hypoxia can induce adaptive responses that complicate the interpretation of hypoxia as purely detrimental. For example, Kucharzewska et al. [169] demonstrated that exosomes secreted by glioblastoma cells under hypoxic conditions promote angiogenesis through intercellular communication, suggesting that tumors may adapt to low oxygen levels via this mechanism. Similarly, Li et al. investigated vasculogenic mimicry—a phenomenon related to hypoxia—where tumors create a blood supply independent of endothelial cells. This process enhances tumor invasiveness and metastasis and is associated with poor prognosis, but it also highlights the remarkable plasticity of tumor cells under hypoxic stress [170]. Furthermore, some researchers have identified seven hypoxia-related genes to construct a prognostic model, which was validated using datasets to effectively predict survival outcomes. Notably, significant differences were observed in immune microenvironment indicators between high-risk and low-risk groups with distinct hypoxia response patterns. Thus, varying hypoxia responses ultimately lead to substantially different prognostic outcomes in gastric cancer patients by altering the tumor immune landscape [171]. In summary, the impact of tumor hypoxia on patient prognosis is highly complex, shaped by a multitude of biological factors and exhibiting diverse, sometimes paradoxical, relationships.

Overall, the relationship between tumor hypoxia and prognosis is multifaceted. The roles of LDH-5, HIF-1α, TRIB3, and hypoxia-related lncRNAs highlight the complex connections between cellular responses to low oxygen levels and cancer progression, underscoring the need for further investigation into these factors as potential therapeutic targets or prognostic indicators. Armed with knowledge of these intricate molecular mechanisms, clinical research aimed at translating this understanding into effective therapies is crucial. To support this effort, we have compiled an overview of existing hypoxia-targeted therapy clinical trials (https://www.clinicaltrials.gov) to provide a comprehensive view of the current research landscape. Table 3 summarizes clinical trials of hypoxia-targeting drugs for cancer treatment. These trials leverage hypoxia tracers and medications that target HIF and its downstream pathways. Hypoxia tracers enable visualization and quantification of tumor hypoxia, allowing clinicians to conduct non-invasive detection and assessment of tumor oxygenation levels. Through PET imaging, these tracers can also monitor dynamic therapeutic responses, potentially facilitating individualized treatment strategies. Medications targeting HIF and its downstream genes can effectively impede tumor growth, improve patient survival rates, and exhibit favorable tolerability. However, these therapies are associated with certain toxicities and side effects. Common adverse events include headache and fatigue, while more severe events, such as gastrointestinal bleeding and thrombosis, have also been reported. Additionally, some clinical trials lack sufficient data to fully assess drug safety and efficacy [32].

## Conclusion

Solid tumors typically experience hypoxia, characterized by significantly lower oxygen levels within cancer cells compared to normal tissues. Even in highly vascularized tumors such as lung cancer, the oxygenation rate remains as low as 2%. This rate can drop even further, particularly in pancreatic cancers, where it may reach as little as 0.3% [50]. Hypoxia plays a crucial role in promoting tumor development, making it a central focus of cancer biology research. This review explores the multifaceted impacts of hypoxia on tumors, including its causes, classification, and the methods used to identify hypoxic regions. Furthermore, we extensively analyze how the hypoxic environment promotes tumor progression, including effects on tumor cell proliferation, immune evasion, and CSC expansion. We summarize the substantial research findings in this field, providing critical insights into tumor biology and informing the development of future therapeutic strategies. Looking ahead, we encourage further exploration of the mechanisms underlying the interaction between hypoxia and tumors, particularly at the molecular and cellular levels. Leveraging newly discovered aspects of hypoxia in cancer treatment—especially through the development of innovative strategies targeting the hypoxic microenvironment—holds great promise. Continued promotion of interdisciplinary collaboration, integrating expertise from biology, medicine, engineering, and other fields, will be essential for achieving a comprehensive understanding and effective management of tumor hypoxia.

## Data Availability

No data was used for the research described in the article.

## References

[1] Ganesh K, Massagué J (2021). Targeting metastatic cancer. Nat Med.

[2] Gerstberger S, Jiang Q, Ganesh K (2023). Metastasis. Cell.

[3] Li YL, Hung WC (2022). Reprogramming of sentinel lymph node microenvironment during tumor metastasis. J Biomed Sci.

[4] Siegel RL, Miller KD, Fuchs HE, Jemal A (2022). Cancer statistics, 2022. CA Cancer J Clin.

[5] Wang JJ, Lei KF, Han F (2018). Tumor microenvironment: recent advances in various cancer treatments. Eur Rev Med Pharmacol Sci.

[6] Anderson NM, Simon MC (2020). The tumor microenvironment. Curr Biol.

[7] Yuan Z, Li Y, Zhang S, Wang X, Dou H, Yu X (2023). Extracellular matrix remodeling in tumor progression and immune escape: from mechanisms to treatments. Mol Cancer.

[8] Kim HJ, Ji YR, Lee YM (2022). Crosstalk between angiogenesis and immune regulation in the tumor microenvironment. Arch Pharm Res.

[9] Jing X, Yang F, Shao C, Wei K, Xie M, Shen H (2019). Role of hypoxia in cancer therapy by regulating the tumor microenvironment. Mol Cancer.

[10] Casazza A, Di Conza G, Wenes M, Finisguerra V, Deschoemaeker S, Mazzone M (2014). Tumor stroma: a complexity dictated by the hypoxic tumor microenvironment. Oncogene.

[11] Tinganelli W, Durante M (2020 Dec 16). Tumor hypoxia and circulating tumor cells. Int J Mol Sci.

[12] Roma-Rodrigues C, Mendes R, Baptista PV, Fernandes AR (2019 Feb 15). Targeting tumor microenvironment for cancer therapy. Int J Mol Sci.

[13] Birner P, Schindl M, Obermair A, Plank C, Breitenecker G, Oberhuber G (2000). Overexpression of hypoxia-inducible factor 1alpha is a marker for an unfavorable prognosis in early-stage invasive cervical cancer. Cancer Res.

[14] Cantelmo AR, Conradi LC, Brajic A, Goveia J, Kalucka J, Pircher A (2016). Inhibition of the glycolytic activator PFKFB3 in endothelium induces tumor vessel normalization, impairs metastasis, and improves chemotherapy. Cancer Cell.

[15] Hosaka K, Yang Y, Seki T, Du Q, Jing X, He X (2020). Therapeutic paradigm of dual targeting VEGF and PDGF for effectively treating FGF-2 off-target tumors. Nat Commun.

[16] Folkman J (1995). Angiogenesis in cancer, vascular, rheumatoid and other disease. Nat Med.

[17] Schito L, Semenza GL (2016). Hypoxia-inducible factors: master regulators of cancer progression. Trends Cancer.

[18] Schito L, Rey S (2017). Hypoxic pathobiology of breast cancer metastasis. Biochim Biophys Acta Rev Cancer.

[19] Horsman MR, Mortensen LS, Petersen JB, Busk M, Overgaard J (2012). Imaging hypoxia to improve radiotherapy outcome. Nat Rev Clin Oncol.

[20] Lee P, Chandel NS, Simon MC (2020). Cellular adaptation to hypoxia through hypoxia inducible factors and beyond. Nat Rev Mol Cell Biol.

[21] Mizrachi A, Ben-Aharon I, Li H, Bar-Joseph H, Bodden C, Hikri E (2021). Chemotherapy-induced acute vascular injury involves intracellular generation of ROS via activation of the acid sphingomyelinase pathway. Cell Signal.

[22] Guo X, Osouli S, Shahripour RB (2023). Review of cerebral radiotherapy-induced vasculopathy in pediatric and adult patients. Adv Biol (Weinh).

[23] Vaupel P, Mayer A (2005). Hypoxia and anemia: effects on tumor biology and treatment resistance. Transfus Clin Biol.

[24] Vaupel P, Mayer A, Briest S, Höckel M (2005). Hypoxia in breast cancer: role of blood flow, oxygen diffusion distances, and anemia in the development of oxygen depletion. Adv Exp Med Biol.

[25] Malmberg P, Hedenström H, Fridriksson HV (1987). Reference values for gas exchange during exercise in healthy nonsmoking and smoking men. Bull Eur Physiopathol Respir.

[26] Blom H, Mulder M, Verweij W (1988). Arterial oxygen tension and saturation in hospital patients: effect of age and activity. BMJ.

[27] Yang J, Harris AL, Davidoff AM (2018 Jan 13). Hypoxia and hormone-mediated pathways converge at the histone demethylase KDM4B in cancer. Int J Mol Sci.

[28] Lee WS, Yang H, Chon HJ, Kim C (2020). Combination of anti-angiogenic therapy and immune checkpoint blockade normalizes vascular-immune crosstalk to potentiate cancer immunity. Exp Mol Med.

[29] Vaupel P, Mayer A (2014). Hypoxia in tumors: pathogenesis-related classification, characterization of hypoxia subtypes, and associated biological and clinical implications. Adv Exp Med Biol.

[30] Baumann R, Depping R, Delaperriere M, Dunst J (2016). Targeting hypoxia to overcome radiation resistance in head & neck cancers: real challenge or clinical fairytale?. Expert Rev Anticancer Ther.

[31] Gamal-Eldeen AM, Fahmy CA, Raafat BM, Althobaiti F, Bassyouni IH, Talaat RM (2022). Circulating levels of hypoxia-regulating MicroRNAs in systemic lupus erythematosus patients with hemolytic anemia. Curr Med Sci.

[32] Chen Z, Han F, Du Y, Shi H, Zhou W (2023). Hypoxic microenvironment in cancer: molecular mechanisms and therapeutic interventions. Signal Transduct Target Ther.

[33] Saxena K, Jolly MK (2019 Aug 3). Acute vs. chronic vs. cyclic hypoxia: their differential dynamics, molecular mechanisms, and effects on tumor progression. Biomolecules.

[34] Liu Q, Palmgren VAC, Danen EH, Le Dévédec SE (2022). Acute vs. chronic vs. intermittent hypoxia in breast cancer: a review on its application in in vitro research. Mol Biol Rep.

[35] Bayer C, Shi K, Astner ST, Maftei CA, Vaupel P (2011). Acute versus chronic hypoxia: why a simplified classification is simply not enough. Int J Radiat Oncol Biol Phys.

[36] Sluimer JC, Daemen MJ (2009). Novel concepts in atherogenesis: angiogenesis and hypoxia in atherosclerosis. J Pathol.

[37] Gupta N, Zhao YY, Evans CE (2019). The stimulation of thrombosis by hypoxia. Thromb Res.

[38] Cooper S (2022). Transferrin-induced thrombosis in hypoxia. Blood.

[39] Batchelor TT, Gerstner ER, Emblem KE, Duda DG, Kalpathy-Cramer J, Snuderl M (2013). Improved tumor oxygenation and survival in glioblastoma patients who show increased blood perfusion after cediranib and chemoradiation. Proc Natl Acad Sci U S A.

[40] Gupta N, Ashraf MZ (2018). Hypoxia signaling in cardiovascular diseases. In: Hypoxia and anoxia London: IntechOpen;.

[41] Bayer C, Vaupel P (2012). Acute versus chronic hypoxia in tumors: Controversial data concerning time frames and biological consequences. Strahlenther Onkol.

[42] Vaupel P, Harrison L (2004). Tumor hypoxia: causative factors, compensatory mechanisms, and cellular response. Oncologist.

[43] Vaupel P, Kelleher DK, Höckel M (2001). Oxygen status of malignant tumors: pathogenesis of hypoxia and significance for tumor therapy. Semin Oncol.

[44] Vaupel P, Thews O, Hoeckel M (2001). Treatment resistance of solid tumors: role of hypoxia and anemia. Med Oncol.

[45] Gaspar BL, Sharma P, Das R (2015). Anemia in malignancies: pathogenetic and diagnostic considerations. Hematology.

[46] Bohlius J, Bohlke K, Castelli R, Djulbegovic B, Lustberg MB, Martino M (2019). Management of cancer-associated anemia with erythropoiesis-stimulating agents: ASCO/ASH clinical practice guideline update. Blood Adv.

[47] Overgaard J, Hoff CM, Hansen HS, Specht L, Overgaard M, Lassen P (2018). DAHANCA 10—effect of darbepoetin alfa and radiotherapy in the treatment of squamous cell carcinoma of the head and neck. a multicenter, open-label, randomized, phase 3 trial by the Danish head and neck cancer group. Radiother Oncol.

[48] van der Heijden M, de Jong MC, Verhagen CVM, de Roest RH, Sanduleanu S, Hoebers F (2019 Apr 25). Acute hypoxia profile is a stronger prognostic factor than chronic hypoxia in advanced stage head and neck cancer patients. Cancers (Basel).

[49] Li K, Yang Y, Ma M, Lu S, Li J (2023). Hypoxia-based classification and prognostic signature for clinical management of hepatocellular carcinoma. World J Surg Oncol.

[50] Muz B, de la Puente P, Azab F, Azab AK (2015). The role of hypoxia in cancer progression, angiogenesis, metastasis, and resistance to therapy. Hypoxia (Auckl).

[51] Thomlinson RH, Gray LH (1955). The histological structure of some human lung cancers and the possible implications for radiotherapy. Br J Cancer.

[52] Bergsjo P, Evans JC (1968). Oxygen tension of cervical carcinoma during the early phase of external irradiation. I. measurements with a Clark micro electrode. Scand J Clin Lab Invest Suppl.

[53] Busk M, Overgaard J, Horsman MR (2020). Imaging of tumor hypoxia for radiotherapy: current status and future directions. Semin Nucl Med.

[54] Wong RK, Fyles A, Milosevic M, Pintilie M, Hill RP (1997). Heterogeneity of polarographic oxygen tension measurements in cervix cancer: an evaluation of within and between tumor variability, probe position, and track depth. Int J Radiat Oncol Biol Phys.

[55] Fleming IN, Manavaki R, Blower PJ, West C, Williams KJ, Harris AL (2015). Imaging tumour hypoxia with positron emission tomography. Br J Cancer.

[56] Panek R, Welsh L, Dunlop A, Wong KH, Riddell AM, Koh DM (2016). Repeatability and sensitivity of T2* measurements in patients with head and neck squamous cell carcinoma at 3T. J Magn Reson Imaging.

[57] Xue F, Chen J, Chen H (2020). Design strategy of optical probes for tumor hypoxia imaging. Sci China Life Sci.

[58] Cheng MHY, Mo Y, Zheng G (2021). Nano versus molecular: optical imaging approaches to detect and monitor tumor hypoxia. Adv Healthc Mater.

[59] Nyayapathi N, Xia J (2019). Photoacoustic imaging of breast cancer: a mini review of system design and image features. J Biomed Opt.

[60] Nasri D, Manwar R, Kaushik A, Er EE, Avanaki K (2023). Photoacoustic imaging for investigating tumor hypoxia: a strategic assessment. Theranostics.

[61] Horsman MR (1998). Measurement of tumor oxygenation. Int J Radiat Oncol Biol Phys.

[62] Bussink J, Kaanders JH, van der Kogel AJ (2003). Tumor hypoxia at the micro-regional level: clinical relevance and predictive value of exogenous and endogenous hypoxic cell markers. Radiother Oncol.

[63] Liu Y, Gu Y, Yuan W, Zhou X, Qiu X, Kong M (2020). Quantitative mapping of liver hypoxia in living mice using time-resolved wide-field phosphorescence lifetime imaging. Adv Sci (Weinh).

[64] Shahpouri M, Adili-Aghdam MA, Mahmudi H, Jaymand M, Amoozgar Z, Akbari M (2023). Prospects for hypoxia-based drug delivery platforms for the elimination of advanced metastatic tumors: from 3D modeling to clinical concepts. J Control Release.

[65] Pries AR, Cornelissen AJ, Sloot AA, Hinkeldey M, Dreher MR, Höpfner M (2009). Structural adaptation and heterogeneity of normal and tumor microvascular networks. PLoS Comput Biol.

[66] Kumar P, Lacroix M, Dupré P, Arslan J, Fenou L, Orsetti B (2024 Jun 14). Deciphering oxygen distribution and hypoxia profiles in the tumor microenvironment: a data-driven mechanistic modeling approach. Phys Med Biol.

[67] Rampling R, Cruickshank G, Lewis AD, Fitzsimmons SA, Workman P (1994). Direct measurement of pO2 distribution and bioreductive enzymes in human malignant brain tumors. Int J Radiat Oncol Biol Phys.

[68] Vaupel P, Schlenger K, Knoop C, Höckel M (1991). Oxygenation of human tumors: evaluation of tissue oxygen distribution in breast cancers by computerized O2 tension measurements. Cancer Res.

[69] Vaupel P, Thews O, Mayer A, Höckel S, Höckel M (2002). Oxygenation status of gynecologic tumors: what is the optimal hemoglobin level?. Strahlenther Onkol.

[70] Höckel M, Schlenger K, Knoop C, Vaupel P (1991). Oxygenation of carcinomas of the uterine cervix: evaluation by computerized O2 tension measurements. Cancer Res.

[71] Gabalski EC, Adam M, Pinto H, Brown JM, Bloch DA, Terris DJ (1998). Pretreatment and midtreatment measurement of oxygen tension levels in head and neck cancers. Laryngoscope.

[72] Terris DJ, Dunphy EP (1994). Oxygen tension measurements of head and neck cancers. Arch Otolaryngol Head Neck Surg.

[73] Becker A, Stadler P, Lavey RS, Hänsgen G, Kuhnt T, Lautenschläger C (2000). Severe anemia is associated with poor tumor oxygenation in head and neck squamous cell carcinomas. Int J Radiat Oncol Biol Phys.

[74] Lartigau E, Randrianarivelo H, Avril MF, Margulis A, Spatz A, Eschwège F (1997). Intratumoral oxygen tension in metastatic melanoma. Melanoma Res.

[75] Le QT, Chen E, Salim A, Cao H, Kong CS, Whyte R (2006). An evaluation of tumor oxygenation and gene expression in patients with early stage non-small cell lung cancers. Clin Cancer Res.

[76] Koong AC, Mehta VK, Le QT, Fisher GA, Terris DJ, Brown JM (2000). Pancreatic tumors show high levels of hypoxia. Int J Radiat Oncol Biol Phys.

[77] Movsas B, Chapman JD, Hanlon AL, Horwitz EM, Pinover WH, Greenberg RE (2001). Hypoxia in human prostate carcinoma: an Eppendorf PO2 study. Am J Clin Oncol.

[78] Mattern J, Kallinowski F, Herfarth C, Volm M (1996). Association of resistance-related protein expression with poor vascularization and low levels of oxygen in human rectal cancer. Int J Cancer.

[79] Lawrentschuk N, Poon AM, Foo SS, Putra LG, Murone C, Davis ID (2005). Assessing regional hypoxia in human renal tumours using 18F-fluoromisonidazole positron emission tomography. BJU Int.

[80] Vaupel P, Mayer A, Höckel M (2006). Oxygenation status of primary and recurrent squamous cell carcinomas of the vulva. Eur J Gynaecol Oncol.

[81] Akakura N, Kobayashi M, Horiuchi I, Suzuki A, Wang J, Chen J (2001). Constitutive expression of hypoxia-inducible factor-1alpha renders pancreatic cancer cells resistant to apoptosis induced by hypoxia and nutrient deprivation. Cancer Res.

[82] Gordan JD, Thompson CB, Simon MC (2007). HIF, c-Myc: sibling rivals for control of cancer cell metabolism and proliferation. Cancer Cell.

[83] Gao N, Nester RA, Sarkar MA (2004). 4-hydroxy estradiol but not 2-hydroxy estradiol induces expression of hypoxia-inducible factor 1alpha and vascular endothelial growth factor a through phosphatidylinositol 3-kinase/Akt/FRAP pathway in OVCAR-3 and A2780-CP70 human ovarian carcinoma cells. Toxicol Appl Pharmacol.

[84] Zhong XS, Zheng JZ, Reed E, Jiang BH (2004). SU5416 inhibited VEGF and HIF-1alpha expression through the PI3K/AKT/p70S6K1 signaling pathway. Biochem Biophys Res Commun.

[85] Hsu JT, Le PH, Lin CJ, Chen TH, Kuo CJ, Chiang KC (2017). Mechanism of salutary effects of melatonin-mediated liver protection after trauma-hemorrhage: p38 MAPK-dependent iNOS/HIF-1α pathway. Am J Physiol Gastrointest Liver Physiol.

[86] LaGory EL, Giaccia AJ (2016). The ever-expanding role of HIF in tumour and stromal biology. Nat Cell Biol.

[87] van Uden P, Kenneth NS, Webster R, Müller HA, Mudie S, Rocha S (2011). Evolutionary conserved regulation of HIF-1β by NF-κB. PLoS Genet.

[88] Li Y, Xu Y, Wang R, Li W, He W, Luo X (2020). Expression of Notch-Hif-1α signaling pathway in liver regeneration of rats. J Int Med Res.

[89] Lau MT, Klausen C, Leung PC (2011). E-cadherin inhibits tumor cell growth by suppressing PI3K/Akt signaling via β-catenin-Egr1-mediated PTEN expression. Oncogene.

[90] Chan N, Koch CJ, Bristow RG (2009). Tumor hypoxia as a modifier of DNA strand break and cross-link repair. Curr Mol Med.

[91] Reynolds TY, Rockwell S, Glazer PM (1996). Genetic instability induced by the tumor microenvironment. Cancer Res.

[92] Li CY, Little JB, Hu K, Zhang W, Zhang L, Dewhirst MW (2001). Persistent genetic instability in cancer cells induced by non-DNA-damaging stress exposures. Cancer Res.

[93] Papp-Szabó E, Josephy PD, Coomber BL (2005). Microenvironmental influences on mutagenesis in mammary epithelial cells. Int J Cancer.

[94] Scanlon SE, Glazer PM (2015). Multifaceted control of DNA repair pathways by the hypoxic tumor microenvironment. DNA Repair (Amst).

[95] Niu Y, Lin Z, Wan A, Sun L, Yan S, Liang H (2021). Loss-of-function genetic screening identifies aldolase a as an essential driver for liver cancer cell growth under hypoxia. Hepatology.

[96] Wrann S, Kaufmann MR, Wirthner R, Stiehl DP, Wenger RH (2013). HIF mediated and DNA damage independent histone H2AX phosphorylation in chronic hypoxia. Biol Chem.

[97] García-Venzor A, Mandujano-Tinoco EA, Ruiz-Silvestre A, Sánchez JM, Lizarraga F, Zampedri C (2020). lncMat2B regulated by severe hypoxia induces cisplatin resistance by increasing DNA damage repair and tumor-initiating population in breast cancer cells. Carcinogenesis.

[98] Riffle S, Pandey RN, Albert M, Hegde RS (2017). Linking hypoxia, DNA damage and proliferation in multicellular tumor spheroids. BMC Cancer.

[99] Faubert B, Vincent EE, Griss T, Samborska B, Izreig S, Svensson RU (2014). Loss of the tumor suppressor LKB1 promotes metabolic reprogramming of cancer cells via HIF-1α. Proc Natl Acad Sci USA.

[100] Chen Z, Zuo X, Zhang Y, Han G, Zhang L, Wu J (2018). MiR-3662 suppresses hepatocellular carcinoma growth through inhibition of HIF-1α-mediated Warburg effect. Cell Death Dis.

[101] Fiaschi T, Marini A, Giannoni E, Taddei ML, Gandellini P, De Donatis A (2012). Reciprocal metabolic reprogramming through lactate shuttle coordinately influences tumor-stroma interplay. Cancer Res.

[102] Zwaans BM, Lombard DB (2014). Interplay between sirtuins, MYC and hypoxia-inducible factor in cancer-associated metabolic reprogramming. Dis Model Mech.

[103] Li L, Yang L, Fan Z, Xue W, Shen Z, Yuan Y (2020). Hypoxia-induced GBE1 expression promotes tumor progression through metabolic reprogramming in lung adenocarcinoma. Signal Transduct Target Ther.

[104] Sebestyén A, Kopper L, Dankó T, Tímár J (2021). Hypoxia signaling in cancer: from basics to clinical practice. Pathol Oncol Res.

[105] Semenza GL (2020). The genomics and genetics of oxygen homeostasis. Annu Rev Genomics Hum Genet.

[106] Chen W, Wu P, Yu F, Luo G, Qing L, Tang J (2022 Nov 10). HIF-1α regulates bone homeostasis and angiogenesis, participating in the occurrence of bone metabolic diseases. Cells.

[107] da Motta LL, Ledaki I, Purshouse K, Haider S, De Bastiani MA, Baban D (2017). The BET inhibitor JQ1 selectively impairs tumour response to hypoxia and downregulates CA9 and angiogenesis in triple negative breast cancer. Oncogene.

[108] Cheng J, Yang HL, Gu CJ, Liu YK, Shao J, Zhu R (2019). Melatonin restricts the viability and angiogenesis of vascular endothelial cells by suppressing HIF-1α/ROS/VEGF. Int J Mol Med.

[109] Xu T, Hu X, Yang G, Liu Y, Zhang Q, Yu S (2022). HIF-1alpha/VEGF pathway mediates 1,3,6,8-tetrabromo-9 H-carbazole-induced angiogenesis: a potential vascular toxicity of an emerging contaminant. J Hazard Mater.

[110] Jiang H, Zhao H, Zhang M, He Y, Li X, Xu Y (2022). Hypoxia induced changes of exosome cargo and subsequent biological effects. Front Immunol.

[111] To KKW, Cho WCS (2022). Exosome secretion from hypoxic cancer cells reshapes the tumor microenvironment and mediates drug resistance. Cancer Drug Resist.

[112] Gajos-Michniewicz A, Duechler M, Czyz M (2014). MiRNA in melanoma-derived exosomes. Cancer Lett.

[113] Sruthi TV, Edatt L, Raji GR, Kunhiraman H, Shankar SS, Shankar V (2018). Horizontal transfer of miR-23a from hypoxic tumor cell colonies can induce angiogenesis. J Cell Physiol.

[114] Sun YL, Cai JQ, Liu F, Bi XY, Zhou LP, Zhao XH (2015). Aberrant expression of peroxiredoxin 1 and its clinical implications in liver cancer. World J Gastroenterol.

[115] Muñoz-Nájar UM, Neurath KM, Vumbaca F, Claffey KP (2006). Hypoxia stimulates breast carcinoma cell invasion through MT1-MMP and MMP-2 activation. Oncogene.

[116] Choi JY, Jang YS, Min SY, Song JY (2011). Overexpression of MMP-9 and HIF-1α in breast cancer cells under hypoxic conditions. J Breast Cancer.

[117] Zhao X, Gao S, Ren H, Sun W, Zhang H, Sun J (2014). Hypoxia-inducible factor-1 promotes pancreatic ductal adenocarcinoma invasion and metastasis by activating transcription of the actin-bundling protein fascin. Cancer Res.

[118] Debnath P, Huirem RS, Dutta P, Palchaudhuri S (2022 Jan 28). Epithelial-mesenchymal transition and its transcription factors. Biosci Rep.

[119] Wicks EE, Semenza GL (2022 Jun 1). Hypoxia-inducible factors: cancer progression and clinical translation. J Clin Invest.

[120] Harten SK, Shukla D, Barod R, Hergovich A, Balda MS, Matter K (2009). Regulation of renal epithelial tight junctions by the von Hippel-Lindau tumor suppressor gene involves occludin and claudin 1 and is independent of E-cadherin. Mol Biol Cell.

[121] Lee SL, Rouhi P, Dahl Jensen L, Zhang D, Ji H, Hauptmann G (2009). Hypoxia-induced pathological angiogenesis mediates tumor cell dissemination, invasion, and metastasis in a zebrafish tumor model. Proc Natl Acad Sci U S A.

[122] Elloul S, Vaksman O, Stavnes HT, Trope CG, Davidson B, Reich R (2010). Mesenchymal-to-epithelial transition determinants as characteristics of ovarian carcinoma effusions. Clin Exp Metastasis.

[123] Park JE, Tan HS, Datta A, Lai RC, Zhang H, Meng W (2010). Hypoxic tumor cell modulates its microenvironment to enhance angiogenic and metastatic potential by secretion of proteins and exosomes. Mol Cell Proteom.

[124] Sitkovsky MV, Kjaergaard J, Lukashev D, Ohta A (2008). Hypoxia-adenosinergic immunosuppression: tumor protection by T regulatory cells and cancerous tissue hypoxia. Clin Cancer Res.

[125] Hao NB, Lü MH, Fan YH, Cao YL, Zhang ZR, Yang SM (2012). Macrophages in tumor microenvironments and the progression of tumors. Clin Dev Immunol.

[126] Wrzesinski SH, Wan YY, Flavell RA (2007). Transforming growth factor-beta and the immune response: implications for anticancer therapy. Clin Cancer Res.

[127] Esebanmen GE, Langridge WHR (2017). The role of TGF-beta signaling in dendritic cell tolerance. Immunol Res.

[128] Le QT, Shi G, Cao H, Nelson DW, Wang Y, Chen EY (2005). Galectin-1: a link between tumor hypoxia and tumor immune privilege. J Clin Oncol.

[129] Whiteside TL, Mandapathil M, Schuler P (2011). The role of the adenosinergic pathway in immunosuppression mediated by human regulatory T cells (Treg). Curr Med Chem.

[130] Barsoum IB, Koti M, Siemens DR, Graham CH (2014). Mechanisms of hypoxia-mediated immune escape in cancer. Cancer Res.

[131] Barsoum IB, Hamilton TK, Li X, Cotechini T, Miles EA, Siemens DR (2011). Hypoxia induces escape from innate immunity in cancer cells via increased expression of ADAM10: role of nitric oxide. Cancer Res.

[132] Badrinath S, Dellacherie MO, Li A, Zheng S, Zhang X, Sobral M (2022). A vaccine targeting resistant tumours by dual T cell plus NK cell attack. Nature.

[133] Parry RV, Chemnitz JM, Frauwirth KA, Lanfranco AR, Braunstein I, Kobayashi SV (2005). CTLA-4 and PD-1 receptors inhibit T-cell activation by distinct mechanisms. Mol Cell Biol.

[134] Barsoum IB, Smallwood CA, Siemens DR, Graham CH (2014). A mechanism of hypoxia-mediated escape from adaptive immunity in cancer cells. Cancer Res.

[135] Salman S, Meyers DJ, Wicks EE, Lee SN, Datan E, Thomas AM (2022 May 2). HIF inhibitor 32-134D eradicates murine hepatocellular carcinoma in combination with anti-PD1 therapy. J Clin Invest.

[136] Gabrilovich D (2004). Mechanisms and functional significance of tumour-induced dendritic-cell defects. Nat Rev Immunol.

[137] Khaled YS, Ammori BJ, Elkord E (2013). Myeloid-derived suppressor cells in cancer: recent progress and prospects. Immunol Cell Biol.

[138] Osada T, Chong G, Tansik R, Hong T, Spector N, Kumar R (2008). The effect of anti-VEGF therapy on immature myeloid cell and dendritic cells in cancer patients. Cancer Immunol Immunother.

[139] Clambey ET, McNamee EN, Westrich JA, Glover LE, Campbell EL, Jedlicka P (2012). Hypoxia-inducible factor-1 alpha-dependent induction of FoxP3 drives regulatory T-cell abundance and function during inflammatory hypoxia of the mucosa. Proc Natl Acad Sci U S A.

[140] Wang Q, Liu C, Zhu F, Liu F, Zhang P, Guo C (2010). Reoxygenation of hypoxia-differentiated dentritic cells induces Th1 and Th17 cell differentiation. Mol Immunol.

[141] Palazón A, Martínez-Forero I, Teijeira A, Morales-Kastresana A, Alfaro C, Sanmamed MF (2012). The HIF-1α hypoxia response in tumor-infiltrating T lymphocytes induces functional CD137 (4-1BB) for immunotherapy. Cancer Discov.

[142] Sarkar S, Germeraad WT, Rouschop KM, Steeghs EM, van Gelder M, Bos GM (2013). Hypoxia induced impairment of NK cell cytotoxicity against multiple myeloma can be overcome by IL-2 activation of the NK cells. PLoS One.

[143] Bao S, Wu Q, McLendon RE, Hao Y, Shi Q, Hjelmeland AB (2006). Glioma stem cells promote radioresistance by preferential activation of the DNA damage response. Nature.

[144] Hill RP, Marie-Egyptienne DT, Hedley DW (2009). Cancer stem cells, hypoxia and metastasis. Semin Radiat Oncol.

[145] Li Z, Bao S, Wu Q, Wang H, Eyler C, Sathornsumetee S (2009). Hypoxia-inducible factors regulate tumorigenic capacity of glioma stem cells. Cancer Cell.

[146] Heddleston JM, Li Z, Lathia JD, Bao S, Hjelmeland AB, Rich JN (2010). Hypoxia inducible factors in cancer stem cells. Br J Cancer.

[147] Liu Y, Ren H, Zhou Y, Shang L, Zhang Y, Yang F (2019). The hypoxia conditioned mesenchymal stem cells promote hepatocellular carcinoma progression through YAP mediated lipogenesis reprogramming. J Exp Clin Cancer Res.

[148] Keith B, Simon MC (2007). Hypoxia-inducible factors, stem cells, and cancer. Cell.

[149] Schweisguth F (2004). Regulation of notch signaling activity. Curr Biol.

[150] Kopan R, Ilagan MX (2009). The canonical Notch signaling pathway: unfolding the activation mechanism. Cell.

[151] Blagosklonny MV (2001). Hypoxia-inducible factor: Achilles’ heel of antiangiogenic cancer therapy (review). Int J Oncol.

[152] Harrison L, Blackwell K (2004). Hypoxia and anemia: factors in decreased sensitivity to radiation therapy and chemotherapy?. Oncologist.

[153] Um JH, Kang CD, Bae JH, Shin GG, Kim DW, Kim DW (2004). Association of DNA-dependent protein kinase with hypoxia inducible factor-1 and its implication in resistance to anticancer drugs in hypoxic tumor cells. Exp Mol Med.

[154] Rohwer N, Cramer T (2011). Hypoxia-mediated drug resistance: novel insights on the functional interaction of HIFs and cell death pathways. Drug Resist Updat.

[155] Zandberg DP, Menk AV, Velez M, Normolle D, DePeaux K, Liu A (2021 May). Tumor hypoxia is associated with resistance to PD-1 blockade in squamous cell carcinoma of the head and neck. J Immunother Cancer.

[156] Mortezaee K, Majidpoor J, Kharazinejad E (2023). The impact of hypoxia on tumor-mediated bypassing anti-PD-(L)1 therapy. Biomed Pharmacother.

[157] Ye Y, Hu Q, Chen H, Liang K, Yuan Y, Xiang Y (2019). Characterization of hypoxia-associated molecular features to aid hypoxia-targeted therapy. Nat Metab.

[158] Zhang N, Tan Y, Yan L, Zhang C, Xu M, Guo H (2020). Modulation of tumor hypoxia by pH-responsive liposomes to inhibit mitochondrial respiration for enhancing sonodynamic therapy. Int J Nanomed.

[159] Kyzas PA, Stefanou D, Batistatou A, Agnantis NJ (2005). Hypoxia-induced tumor angiogenic pathway in head and neck cancer: an in vivo study. Cancer Lett.

[160] Koukourakis MI, Giatromanolaki A, Sivridis E, Bougioukas G, Didilis V, Gatter KC (2003). Lactate dehydrogenase-5 (LDH-5) overexpression in non-small-cell lung cancer tissues is linked to tumour hypoxia, angiogenic factor production and poor prognosis. Br J Cancer.

[161] Urano N, Fujiwara Y, Doki Y, Tsujie M, Yamamoto H, Miyata H (2006). Overexpression of hypoxia-inducible factor-1 alpha in gastric adenocarcinoma. Gastric Cancer.

[162] Isobe T, Aoyagi K, Koufuji K, Shirouzu K, Kawahara A, Taira T (2013). Clinicopathological significance of hypoxia-inducible factor-1 alpha (HIF-1α) expression in gastric cancer. Int J Clin Oncol.

[163] Zhang L, Wang S, Wang Y, Zhao W, Zhang Y, Zhang N (2021). Effects of hypoxia in intestinal tumors on immune cell behavior in the tumor microenvironment. Front Immunol.

[164] Kapinova A, Kubatka P, Zubor P, Golubnitschaja O, Dankova Z, Uramova S (2018). The hypoxia-responsive long non-coding RNAs may impact on the tumor biology and subsequent management of breast cancer. Biomed Pharmacother.

[165] Zhao Y, Liu L, Zhao J, Du X, Yu Q, Wu J (2021). Construction and verification of a hypoxia-related 4-lncRNA model for prediction of breast cancer. Int J Gen Med.

[166] Wennemers M, Bussink J, Grebenchtchikov N, Sweep FC, Span PN (2011). TRIB3 protein denotes a good prognosis in breast cancer patients and is associated with hypoxia sensitivity. Radiother Oncol.

[167] Wennemers M, Bussink J, Scheijen B, Nagtegaal ID, van Laarhoven HW, Raleigh JA (2011). Tribbles homolog 3 denotes a poor prognosis in breast cancer and is involved in hypoxia response. Breast Cancer Res.

[168] Chiavarina B, Martinez-Outschoorn UE, Whitaker-Menezes D, Howell A, Tanowitz HB, Pestell RG (2012). Metabolic reprogramming and two-compartment tumor metabolism: opposing role(s) of HIF1α and HIF2α in tumor-associated fibroblasts and human breast cancer cells. Cell Cycle.

[169] Kucharzewska P, Christianson HC, Welch JE, Svensson KJ, Fredlund E, Ringnér M (2013). Exosomes reflect the hypoxic status of glioma cells and mediate hypoxia-dependent activation of vascular cells during tumor development. Proc Natl Acad Sci U S A.

[170] Li S, Meng W, Guan Z, Guo Y, Han X (2016). The hypoxia-related signaling pathways of vasculogenic mimicry in tumor treatment. Biomed Pharmacother.

[171] Guo J, Xing W, Liu W, Liu J, Zhang J, Pang Z (2022). Prognostic value and risk model construction of hypoxic stress-related features in predicting gastric cancer. Am J Transl Res.

